# The role of machine learning in developing non-magnetic resonance imaging based biomarkers for multiple sclerosis: a systematic review

**DOI:** 10.1186/s12911-022-01985-5

**Published:** 2022-09-15

**Authors:** Md Zakir Hossain, Elena Daskalaki, Anne Brüstle, Jane Desborough, Christian J. Lueck, Hanna Suominen

**Affiliations:** 1 School of Computing, College of Engineering and Computer Science, Australian National University, Canberra, ACT Australia; 2 The John Curtin School of Medical Research, College of Health and Medicine, Australian National University, Canberra, ACT Australia; 3 Department of Health Services Research and Policy, Research School of Population Health, College of Health and Medicine, Australian National University, Canberra, ACT Australia; 4 Department of Neurology, Canberra Hospital, Canberra, ACT Australia; 5 ANU Medical School, College of Health and Medicine, Australian National University, Canberra, ACT Australia; 6 Department of Computing, University of Turku, Turku, Finland

**Keywords:** Deep learning, Disease progression, Medical informatics, Multiple sclerosis, Prognosis, Supervised machine learning, Systematic review

## Abstract

**Background:**

*Multiple sclerosis* (MS) is a neurological condition whose symptoms, severity, and progression over time vary enormously among individuals. Ideally, each person living with MS should be provided with an accurate prognosis at the time of diagnosis, precision in initial and subsequent treatment decisions, and improved timeliness in detecting the need to reassess treatment regimens. To manage these three components, discovering an accurate, objective measure of overall disease severity is essential. *Machine learning* (ML) algorithms can contribute to finding such a clinically useful biomarker of MS through their ability to search and analyze datasets about potential biomarkers at scale. Our aim was to conduct a systematic review to determine how, and in what way, ML has been applied to the study of MS biomarkers on data from sources other than magnetic resonance imaging.

**Methods:**

Systematic searches through eight databases were conducted for literature published in 2014–2020 on MS and specified ML algorithms.

**Results:**

Of the 1, 052 returned papers, 66 met the inclusion criteria. All included papers addressed developing classifiers for MS identification or measuring its progression, typically, using hold-out evaluation on subsets of fewer than 200 participants with MS. These classifiers focused on biomarkers of MS, ranging from those derived from omics and phenotypical data (34.5% clinical, 33.3% biological, 23.0% physiological, and 9.2% drug response). Algorithmic choices were dependent on both the amount of data available for supervised ML (91.5%; 49.2% classification and 42.3% regression) and the requirement to be able to justify the resulting decision-making principles in healthcare settings. Therefore, algorithms based on decision trees and support vector machines were commonly used, and the maximum average performance of 89.9% AUC was found in random forests comparing with other ML algorithms.

**Conclusions:**

ML is applicable to determining how candidate biomarkers perform in the assessment of disease severity. However, applying ML research to develop decision aids to help clinicians optimize treatment strategies and analyze treatment responses in individual patients calls for creating appropriate data resources and shared experimental protocols. They should target proceeding from segregated classification of signals or natural language to both holistic analyses across data modalities and clinically-meaningful differentiation of disease.

**Supplementary Information:**

The online version contains supplementary material available at 10.1186/s12911-022-01985-5.

## Background

*Multiple sclerosis *(MS) is a condition affecting the *central nervous system* (CNS) characterised by a mixture of inflammation and neurodegeneration. Several disease patterns (a.k.a. phenotypes) are recognized, including, but not limited to, *relapsing remitting MS* (RRMS) and *secondary progressive MS* (SPMS), but the clinical course varies considerably among individuals [1]. In recent years, the number of treatments available to reduce inflammatory processes has increased dramatically: these agents can be very effective in suppressing clinical disease activity, but they are not effective in all patients and many of them are associated with an appreciable risk of significant side effects. This has resulted in a drive towards personalised treatment for *people living with MS* (PwMS); ideally, individuals should be provided with (i) an accurate prognosis at the time of diagnosis, (ii) optimization of initial treatment decisions, and (iii) greater precision in following up the response to treatment and, therefore, early detection of the need to modify a particular treatment regimen [2].

To manage these three components, it is essential to discover an accurate, objective way of measuring overall disease severity, or status. However, in common with many neurological conditions, MS still lacks such a measure. Diagnosis is based on a combination of clinical features and information obtained from diagnostic tests, most notably *magnetic resonance imaging* (MRI) [3]. Clinical disease severity is generally quantified using the *Expanded Disability Status Scale* (EDSS), *MS Severity Score* (MSSS), or *MS Functional Composite* (MSFC) [4, 5], but these tools have drawbacks: each of them suffers from intra-subject and intra-observer variability and the EDSS and MSSS are biased towards the motor domain [6].

Accordingly, there has been a search for a biomarker of MS that would facilitate more accurate and objective definition of disease severity/status. A biomarker has been defined as “a characteristic that is objectively measured and evaluated as an indicator of normal biological processes, pathogenic processes, or pharmacologic responses to a therapeutic intervention” [7]. MRI is currently the most widely-used biomarker in MS. However, it is not ideal: abnormalities on MRI are not well correlated with clinical manifestations of disease; it is expensive, invasive, and time-consuming; and it requires patients to travel to MRI scanners. Hence, several alternative biomarkers — spanning from blood or breath analysis to cognitive measures — are undergoing assessment in different centres [8–10]. Although this research into a suitable clinical biomarker other than MRI has been extensive, no clear candidate that might complement, or replace, MRI has yet been found.

An effective biomarker of MS would also contribute to better overall health and healthcare experience of PwMS. Research examining the experiences of PwMS describes a lack of information and support, particularly at the time of diagnosis [11, 12], requiring extensive personal effort to meet patients’ information needs during an already stressful time [13]. Experiences of uncertainty dominate this literature, when considering treatment options and possible side effects, and in dealing with the impact of MS on work, family, and social life [14, 15]. Identification of a reliable biomarker would help.

The focus of this systematic review is to study *machine learning* (ML) as a way to support the discovery of biomarkers that can be measured regularly and inexpensively using non-invasive and readily-accessible techniques, thus reducing the test burden on PwMS and optimizing early detection and treatment management. ML refers to computational algorithms for gathering and making sense of evidence derived from large volumes of data thereby permitting, or facilitating, human judgement and decision-making [16, 17] (see Supplementary Material A for further background on ML problems; *supervised* and *unsupervised* ML algorithms; and their timeline). ML has the potential to help in the search for a clinically useful biomarker because it can assess how well candidate biomarkers perform in the assessment of disease severity and prognosis, either individually or in combination. ML may also assist in developing decision-support techniques to aid clinicians and PwMS in making optimal individual treatment choices and in assessing the response to a chosen treatment.

To determine how best to apply ML, it is important to begin by ascertaining what is already known. Comprehensive reviews of ML-assisted MRI analysis in MS have already been performed [18, 19]. However, to date, ML has been applied less frequently to other type of biomarkers [20]. This systematic review was therefore designed to investigate how ML has been applied to the study of potential non-MRI biomarkers in the management of MS, looking specifically at prognosis, disease severity, choice of treatment, and assessment of response to treatment.

## Methods

The present systematic literature review, registered under the international *prospective register of systematic reviews* (PROSPERO) number CRD42020163161, followed the *preferred reporting items for systematic reviews and meta-analyses* (PRISMA) guidelines [21]. Eight resources — PubMed, Cochrane, Google Scholar^1^, ScienceDirect, Scopus, Web of Science, Lens, and dblp — were used as the primary tools for indexing and retrieving publications, granted their index size and retrieval reliability [22]. The search query was formed by combining the term “Multiple Sclerosis” with a number of ML related terms as described in Table 1. Namely, depending on the resource, both general queries and their more specific variants were used to maximize number of returned relevant publications. Papers published over the 5 years following the introduction of *generative adversarial networks* (GANs; Supplementary Material A) [23] (i.e., from 1 January 2014 to 31 January 2020) were considered.

**Table 1:** “Multiple Sclerosis” and specific machine learning algorithms returned 1, 052 studies from eight search resources

Search terms	Search resource	Number of returned studies
“Multiple Sclerosis” AND (“Machine Learning” OR “Machine Intelligence”		
OR “Deep Learning” OR “Decision Tree*” OR “Random Forest*”	PubMed	75
OR “Pattern Recognition” OR “Genetic Algorithm*” OR “Supervised Algorithm*”		
OR “Decision Support System*” OR “Evolutionary Computation*”	Cochrane	25
OR “Neural Network*” OR “Support Vector Machine*” OR “Autoencoder*”	Google scholar	100 #
OR “Deep Belief Network*” OR “Adversarial Network*”		
OR “Self Organizing Map*” OR “Self Organising Map*”)		
“Multiple Sclerosis” AND (“machine learning” OR “machine intelligence”)	Science direct	340 #
	Scopus	169
	Web of Science	179 #
	Lens	160
“Multiple sclerosis” AND “machine learning”	dblp	4 #
Total count (# Sort by relevance)		1052

# Sort by relevance

In order to ensure a low risk of bias, initial searches were conducted by three medical ML researchers. They performed independent searches (Table 1) using the protocol described below and each collected a list of relevant publications. The decision to include or exclude any article not found as relevant by all three reviewers was made through discussion until a consensus was reached.

The following *exclusion criteria* (EC) were defined:

1. Duplicates were removed.
2. Publications that were not original full peer-reviewed papers (e.g., reviews, book chapters, surveys, and abstracts) were removed.
3. Papers that were not about PwMS were removed.
4. Papers that were not about ML were removed.
5. Papers working solely on data from MRI, optical coherence tomography, visual perimetry, and/or lumbar puncture were removed because these examinations are either not routinely conducted as standard clinical tests for MS or were not aligned with our focus on biomarkers that can be measured regularly and inexpensively using minimally invasive and readily-accessible techniques.

**Fig. 1:**
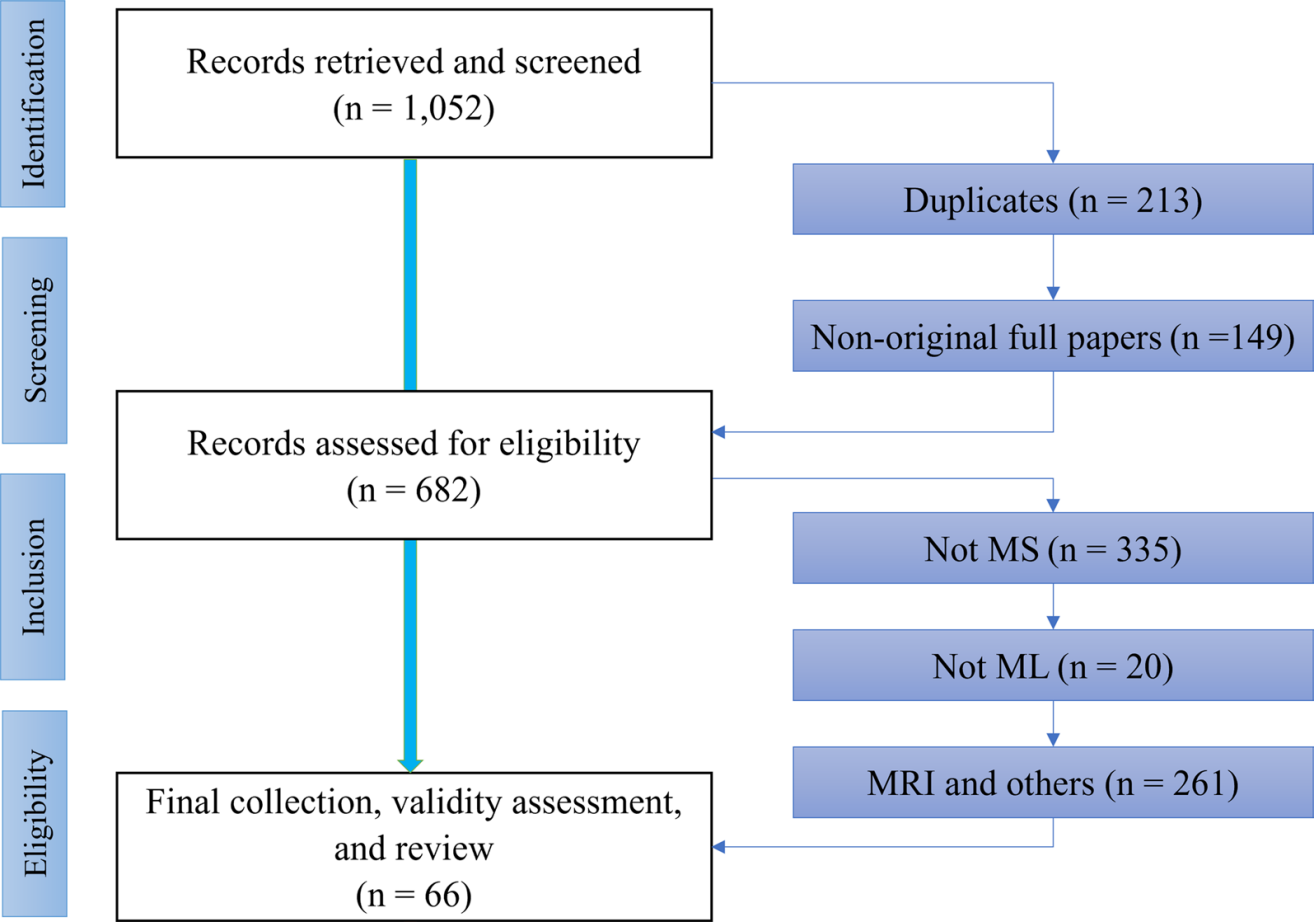
Flow chart of the systematic review process

The selection of the studies considered in this review was performed in four phases (Fig. 1). In the identification phase, the previously discussed search keywords constrained within the search time frame were applied in the databases and resulted in 1, 052 publications. In the screening phase, 368 publications were were excluded as duplicates (EC.1) or non-original papers (EC.2), leaving 682 documents. In the eligibility phase, 355 papers were excluded as they did not consider MS and ML (EC.3 and EC.4). A further 261 papers were excluded on the basis of looking at MRI or other pre-specified tool (EC.5).

**Fig. 2:**
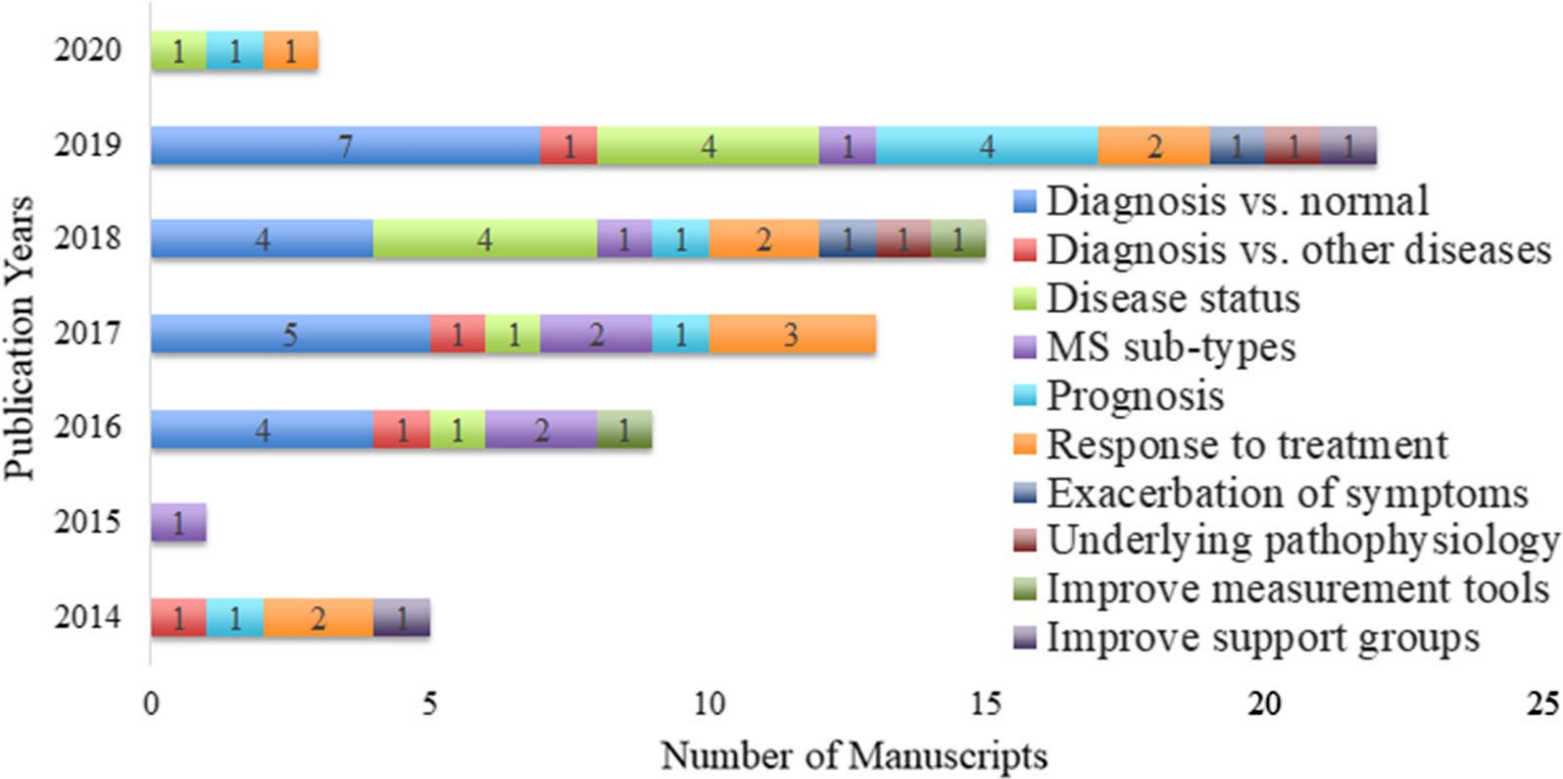
Distribution of manuscripts with publication years. The total number of publications adds up to 68 because out of the 66 included publications, one discussed both diagnosis and MS sub-types and another discussed both diagnosis and prognosis

Ultimately, 66 papers remained for studying; the majority of them ($n = 22$) were published in 2019, followed by 15 and 13 papers in 2018 and 2017, respectively (Fig. 2).

As a validity assurance method, these papers were assessed with respect to the guidelines for developing and reporting ML analyses and predictive models in biomedical and clinical research [24, 25] (see Additional file 2 for the outcomes). Because almost all criteria included in the guidelines were followed, no further exclusions were made.

## Results

**Table 2:** Summary of 49 included papers that reported on applications towards supporting diagnosis, disease status assessment, MS sub-typing, and prognosis. See Table 3 for a summary of 17 included papers that reported on other applications. Abbreviations as below in the Table

Author	Data sources	ML methods	Outcomes
*Diagnosis vs normal*			
Ahmadi et al. [26]	EEG	OS-ELM;	Accuracy in [90.0%, 91.0%].
Andersen et al. [27]	Metabolomics	LR; RF;	AUC in [81.0%, 86.0%].
Bertolazzi et al. [28]	Genes	KNN; SVM; DT;	Accuracy in [92.0%, 95.0%].
Broza et al. [29]	Breath markers	NN;	Accuracy in [72.0%, 90.0%];
			AUC in [79.0%, 87.0%].
Chase et al. [30]	Medical records	NB; NLP;	AUC in [90.0%, 94.0%].
deAndrés-G. et al. [31]	Genetic pathways	Distance-based classifier;	Accuracy in [93.8, 98.2%].
		Minimum spanning tree;	Neurogenesis and Hemoglobin related genes.
Galli et al. [32]	Lymphocytes	NN;	TNF, GM-CSF, IFN-$\gamma$, IL2, and CXCR4.
Goldstein et al. [33]	SNP	RF; LASSO; GLM; KNN; LR;	CRHR1.
Goyal et al. [34]	Cytokines	SVM; NN; DT; RF;	Accuracy = 90.9%; AUC = 95.7%.
Lötsch et al. [35]	Lipid markers	SOM; AdaBoost; KNN; RF;	Accuracy in [92.5%, 100%]; AUC in [92.5%, 100%].
Lötsch et al. [36]	Lipid markers	SOM;	Accuracy in [77.0%, 94.6%]; Ceramides.
Perera et al. [37]	Tremor	Linear Regression; SVR; RF;	Accuracy in [84.2%, 90.8%]; Velocity of index finger.
Prabahar et al. [38]	MicroRNA	SVM;	Accuracy in [87.8%, 90.1%].
Severini et al. [39]	Balance board	SVM;	Accuracy in [83.3, 85.5%].
Telalovic et al. [40]	lncRNAs	RF;	Accuracy in [61.5%, 84.6%].
Torabi et al. [41]	EEG	SVM; KNN;	Accuracy in [79.8%, 93.1%].
Zhang et al. [42]	Genetic pathways	SVM;	Accuracy in [61.2%, 70.3%].
Kiiski et al. [43]	ERPs	Linear Regression;	Visual task is better than auditory task.
Saroukolaei et al. [44]	Enzymes	Linear Regression; NN;	Higher CA.
Sun et al. [45]	Postural sway	RF;	Accuracy in [92.3%, 95.6%].
*Diagnosis vs other diseases*			
Bang et al. [46]	Gut microbial	SVM; KNN; LogitBoost; Logistic Tree;	Accuracy in [96.4%, 98.3%].
Guo et al. [47]	Transcriptomics	KNN; SVM; NB; NN; LR; RF;	Accuracy in [77.2%, 86.4%];
			TNFSF10 is allied to the PwMS.
Ohanian et al. [48]	Key symptoms	DT;	Accuracy in [79.2%, 81.2%];
			Immune domain is useful in this case.
Ostmeyer et al. [49]	B-cell receptor	Optimize Log Likelihood;	Accuracy in [72.0%, 87.0%].
*Disease status*			
Azrour et al. [50]	Gait analysis	DT;	EDSS score in [< 0.97 (No MS), >4.15 (MS)].
Fritz et al. [51]	Falls risk	LR;	Fallers and near-fallers are at similar risks.
Gudesblatt et al. [52]	Falls risk	RF;	Accuracy in [82.9%, 91.2%];
			F1 score in [78.9%, 91.3%].
Haider et al. [53]	Body movements	SVM; KNN; RF;	Accuracy in [95.5%, 100%].
Jackson et al. [54]	Genetic markers	RF;	19 genetic variants.
Kosa et al. [55]	Clinical data, MEP	GA;	CombiWISE is better than MRI measures.
McGinnis et al. [56]	Gait speeds	SVR;	RMSE speed in [0.12 m/s, 0.14 m/s].
Morrison et al. [57]	Motor assessment	DT; SVM;	Visualisation reduce gap between human and ML.
Shahid et al. [58]	Clinical data	KNN; SVM; RF; Rough Set;	Accuracy in [79.7%, 84.0%].
Supratak et al. [59]	Walking speed	SVR;	Walking speed in [0.57 m/s, 1.22 m/s].
*MS sub-types*			
Acquarelli et al. [60]	Pathology	NLP; Clustering;	Pathological profiles and disease duration.
Fiorini et al. [61]	Clinical data	LS; LR; SVM; KNN;	Accuracy in [75.0%, 78.3%];
			F1 score in [62.3%, 70.2%].
Gronsbell et al. [62]	EMR	SSL;	Accuracy in [92.9%, 93.9%].
Gupta et al. [63]	Microbiomics	RF;	Specificity = 86.4%; Sensitivity = 45.4%.
Lim et al. [64]	Kyneurenine	DT; DA; CART; SVM;	Accuracy in [83.0%, 91.0%].
Lopez et al. [65]	Genetic signatures	Clustering;	CD69, CCR5, IL13, and STAT3.
*Prognosis*			
Bejarano et al. [66]	Clinical, MEP	NB; NN; LR; DT; Linear Regression;	Accuracy in [67.0%, 80.0%]; AUC in [65%, 76.0%].
Brichetto et al. [67]	Clinical data	Supervised Algorithms;	Accuracy in [82.6%, 86.0%].
Briggs et al. [68]	Clinical data	LASSO;	Obesity and smoking.
Flauzino et al. [69]	Clinical data	LR; NN;	AUC = 84.2; Lower IL4.
Pruenza et al. [70]	Clinical data	RF;	AUC in [80.0%, 82.0%].
Tacchella et al. [71]	Clinical data	RF;	AUC in [69.6%, 72.5%].
Yperman et al. [72]	MEP	RF; LR;	AUC in [72.0%, 75.0%].
Zhao et al. [73]	Clinical data	SVM; LR;	Accuracy in [68.0%, 73.0%].
Zhao et al. [74]	Clinical data	SVM; KNN; AdaBoost;	Accuracy in [76.0%, 90.0%].

**Fig. 3:**
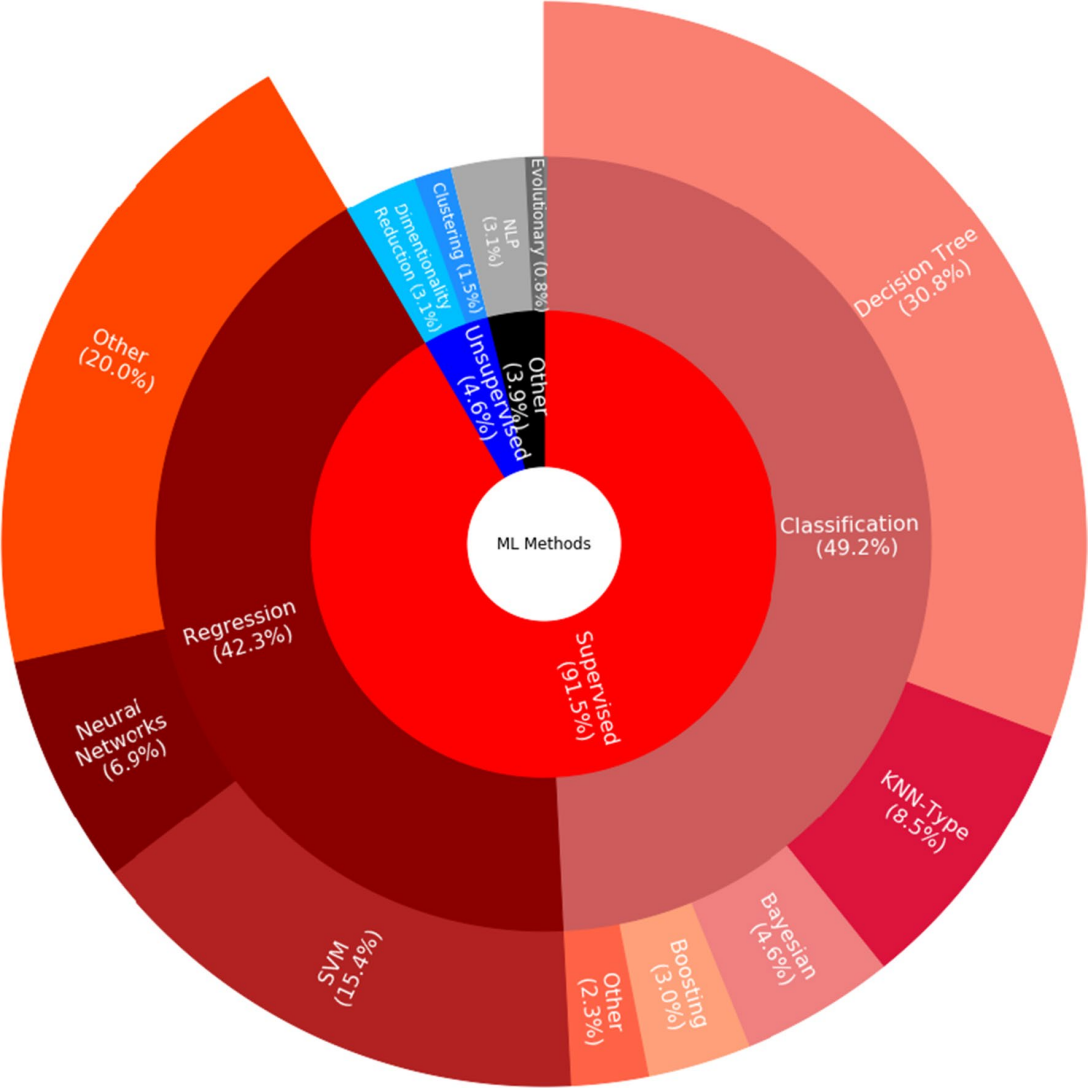
Sunburst chart of machine learning algorithms applicable to multiple sclerosis studies

**Fig. 4:**
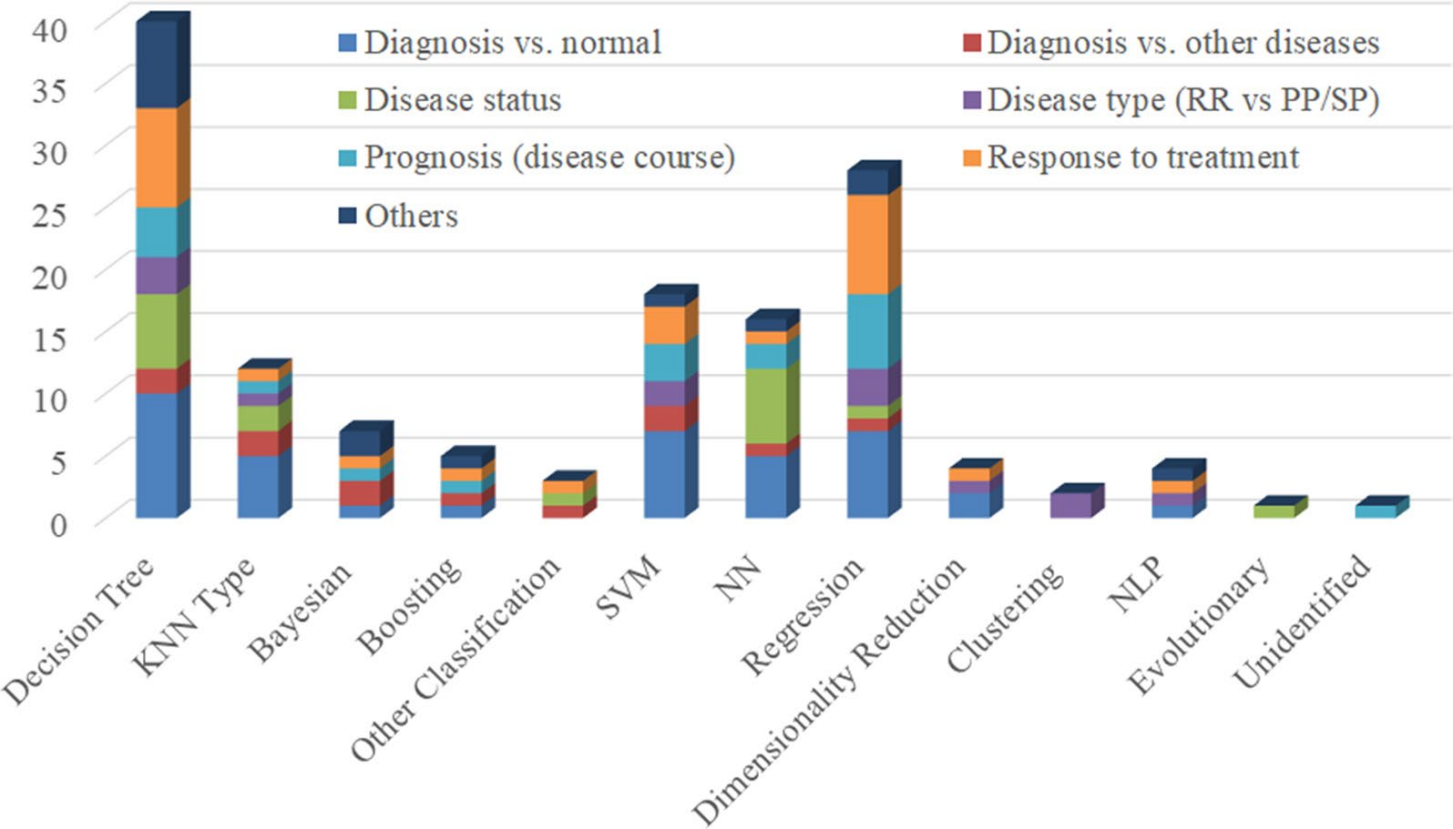
Histogram of machine learning algorithms in multiple sclerosis studies. The *y*-axis refers to the number of studies

**Fig. 5:**
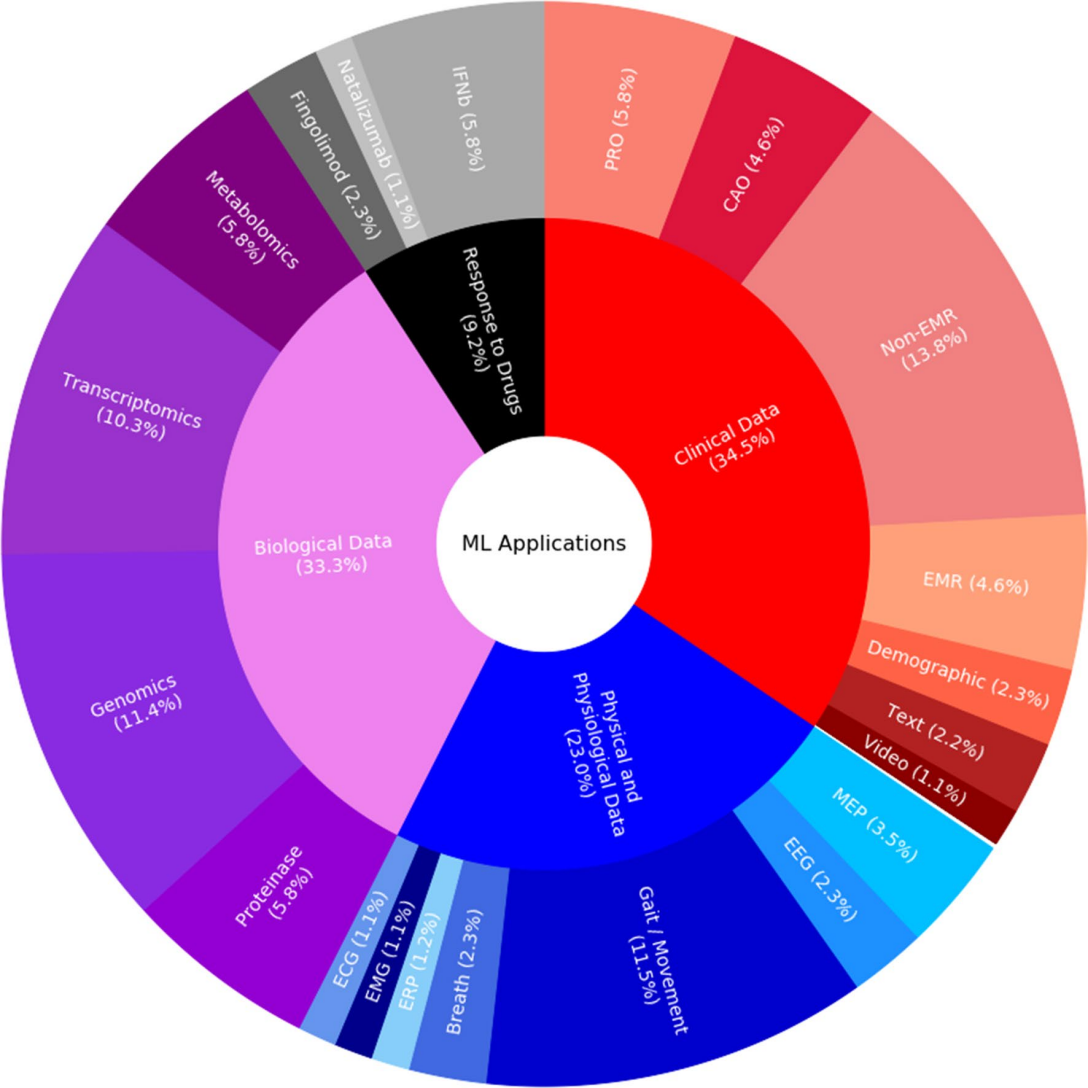
Sunburst chart of machine learning applications and data in multiple sclerosis studies

**Fig. 6:**
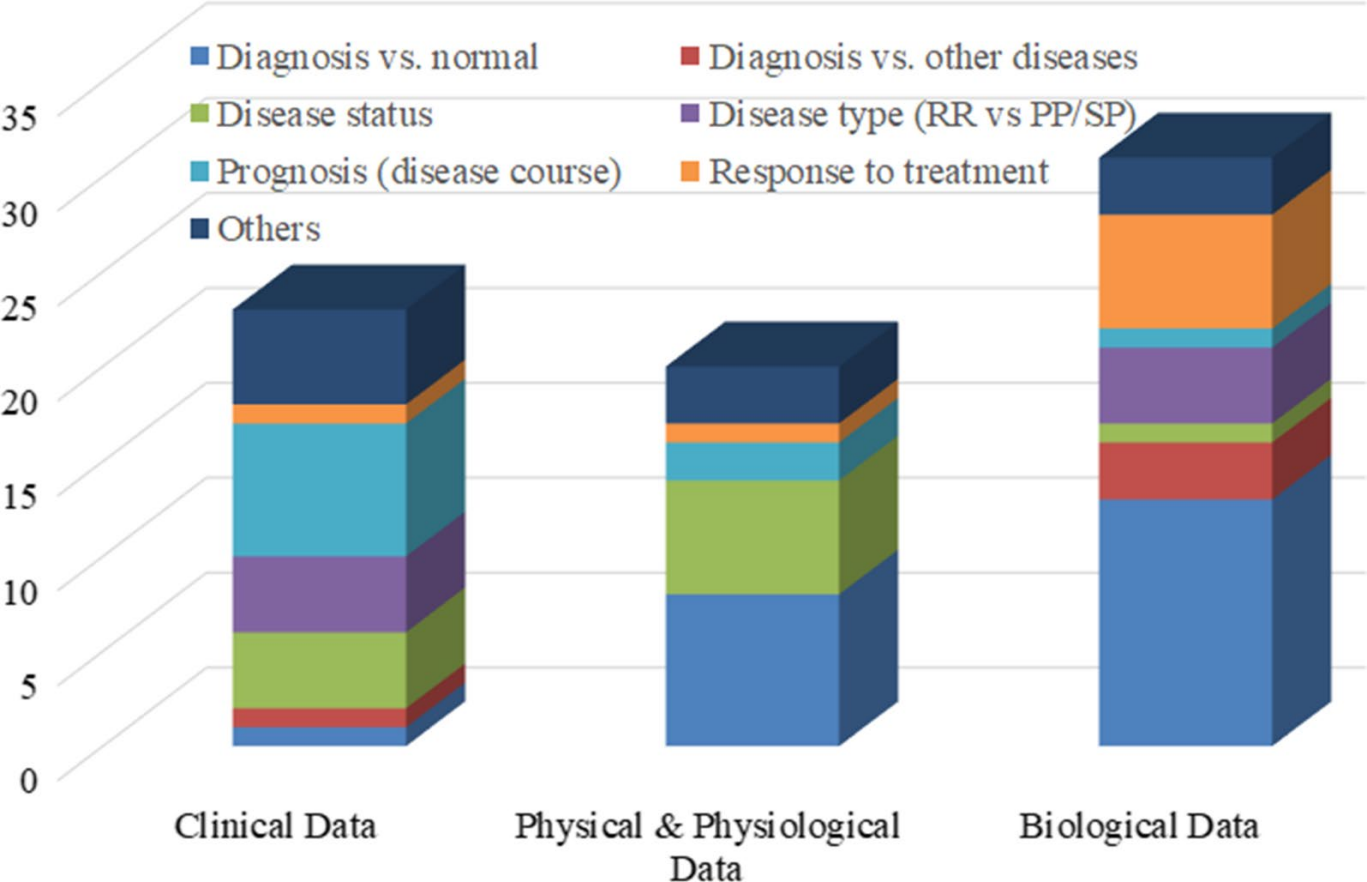
Histogram of data for ML applications. The *y*-axis refers to the number of studies

**Measures:** Accuracy = (TP + TN) / (TP + TN + FP + FN); FPR =FP(FP+TN); Precision = TP / (TP+FP); F1 Score = 2*(Recall * Precision) / (Recall + Precision); Sensitivity / Recall / TPR = TP / (TP + FN); Specificity = TN / (TN + FP); AUC = Area Under the ROC curve, calculated from the plot of TPR vs. FPR;

**Technical:** CART = Classification and Regression Tree; DA = Discriminant Analysis; DT = Decision Tree; ET = Extra-Trees; FN = False Negatives; FP = False Positives; FPR = False Positive Rate; GA = Genetic Algorithm; GAIMS = Gait Analysis Imaging System; GB = Gradient Boosting; GLM = Generalized Linear Model; IP-GRASP = A Greedy Randomized Adaptive Search Procedure with memory; IRT = Item Response Theory; KNN = k-nearest Neighbour; LASSO = Least absolute shrinkage and selection operator; LR = Logistic Regression; LS = Least Squares; ML = Machine Learning; MRI = Magnetic Resonance Imaging; NB = Naïve Bayes; NLP = Natural Language Processing; NN = Neural Network; OS-ELM = Online Sequential Extreme Learning Machine; QoL = Quality of Life; RF = Random Forest; RMSE = Root Mean Square Error; ROC = Receiver Operating Characteristic; RR = Relapsing-Remitting Multiple Sclerosis; SC = Shrunken Centroid; SOM = Self-Organising Map; SNAc = Social Network Analysis-based Classifier; SSL = Semi-supervised Learning; SVM = Support Vector Machines; TN = True Negatives; TP = True Positives; TPR = True Positive Rate;

**Biomedical:** CA = Candida Albicans; CAO = Clinician Assessed Outcomes; CFS = Chronic Fatigue Syndrome; CIS = Clinically Isolated Syndrome; EDSS = Expanded Disability Status Scale; EEG = Electroencephalogram; EMG = Electromyogram; EMR = Electronic Medical Record; ERPs = Event Related Potentials; HC = Healthy Controls; IM &NO = Immune-inflammatory, Metabolic, and Nitro-Oxidative; KP = Kynurenine Pathway; lncRNAs = long non-coding RNAs; ME = Myalgic Encephalomyelitis; MEP = Motor Evoked Potentials; MS = Multiple Sclerosis; NAb = Neutralising Antibodies; PP = Primary-Progressive Multiple Sclerosis; PRO = Patient Reported Outcomes; PwMS = people living with MS; rRNA = Ribosomal Ribonucleic Acid; SP = Secondary-Progressive Multiple Sclerosis; without MS = people living without Multiple Sclerosis; WE = Word Embedding;

**Genetics:** C6ORF10 = Chromosome 6 Open Reading Frame 10; CASP2 = Caspase 2, Apoptosis-Related Cysteine Peptidase; CCR5 = C-C Chemokine Receptor Type 5; CD69 = CD69 Antigen (P60, Early T-Cell Activation Antigen); CRHR1 = Corticotropin Releasing Hormone Receptor 1; CXCR4 = C-X-C Motif Chemokine Receptor 4; GM-CSF = Granulocyte-Macrophage Colony-Stimulating Factor; HLA-DRB1 = Human Leukocyte Antigen haplotype, DR beta 1; IFN-$\beta$ = Interferon beta; IFN-$\gamma$ = Interferon Gamma; IL2 = Interleukin 2, T Cell Growth Factor; IL4 = Interleukin 4; IL10 = Interleukin 10; IL12Rb1 = Interleukin 12 Receptor Subunit Beta 1; IL13 = Interleukin 13; TAP2 = Transporter 2, ATP Binding Cassette Subfamily B Member; TNF = Tumor Necrosis Factor; TNFSF10 = Tumor Necrosis Factor (ligand) superfamily, member 10; STAT3 = Signal Transducer and Activator Of Transcription 3;

The 66 included studies explored the application of ML to MS for purposes ranging from diagnosis and prognosis to measuring disease status and severity levels (Tables 2 and 3; Additional file 3). They all followed the recommended reporting guidelines [24, 25] from what to include when reporting predictive models in biomedical research to how to succinctly present standardized results of ML methods. In these studies, algorithmic choices were dependent on both the amount of data available for supervised ML and the requirement to be able to justify the resulting decision-making principles in healthcare settings. Typically, datasets with fewer than 200 PwMS were available for supervised ML and, therefore, *support vector machines* (SVMs) and decision tree-based algorithms were common (Figs. 3 and 4; Additional file 1). These ML applications focused on biomarkers of MS, ranging from those derived from omics and phenotypical data (e.g., cognitive, balance, gait, or other clinical tests) to patients’ self-reported assessments (Figs. 5 and 6).

### Aims and outcomes of applications

ML applications to differentiate PwMS from controls emphasized the benefits of a diversity of data sources in the search for a clinically useful biomarker of MS (Table 2 and Additional file 3). This differentiation problem was studied in as many as 20 out of the 66 included studies ($30.3\%$) [26–45]. These experiments claimed an accuracy of over 90.0% in ML looking at medical records [30], *electroencephalogram* (EEG) signals [26, 41], tremor or postural-sway measurements [37, 45], and omics data [28, 34–36, 38]. Decision trees [28, 34], random (decision) forests [34, 35, 37, 45], SVMs [28, 34, 37, 38, 41], *neural networks* (NNs) [26, 34], *self-organizing maps* (SOMs) [35, 36], and the naïve Bayes algorithm [30] resulted in the best learning performance. Analyzing the contribution of data sources, modalities, and featurizations to the ML performance, studies [32, 33, 36, 37, 44] supported the possibility of measuring and evaluating stress, anxiety, depression, obesity, and/or inflammatory markers^2^ as diagnostic biomarkers of MS.

**Table 3:** Summary of the included papers that reported on applications towards evaluating response to treatment, symptoms, or underlying pathophysiology together with those for improving measurement tools or support groups. Abbreviations as above in Table 2

Author	Data sources	ML methods	Outcomes
*Response to treatment*			
Baranzini et al. [75]	INF-$\beta$ response	RF;	Accuracy in [75.0%, 82.0%];
			CASP2 / IL10 / IL12Rb1.
Ebrahimkhani et al. [76]	microRNA	LR; RF;	AUC in [65.2%, 91.1%].
Fagone et al. [77]	Genomics	UCSC;	Accuracy = 89.2%.
Karim et al. [78]	INF-$\beta$ response	CART; LASSO; SVM; LR;	Hazard Ratio[4] in [1.359, 1.372].
Kasatkin et al. [79]	Flu-like symptoms	NN; Static Model;	Sensitivity in [73.4%, 81.2%];
			Specificity in [71.6%, 80.6%].
Li et al. [80]	Cardiac data	DT;	Baseline hare rate (HR).
Üçer et al. [81]	INF-$\beta$ response	SNAc; SVM; KNN; RF; NB; LR; DT;	Accuracy in [63.1%, 64.5%];
			F1 score in [77.4%, 78.3%];
Walter et al. [82]	Costing data	DT;	NAb is cheaper than other tests.
Patrick et al. [83]	RNAs	GB; LR; RF; LASSO; DA; Nearest SC; WE;	AUC in [72.1%, 89.9%];
*Exacerbation of symptoms*			
Bhattacharya et al. [84]	Daily activities	NN;	Fatigue.
Papakostas et al. [85]	EMG	SVM; RF; ET; Gradient-Boosting;	F1 Score in [75.1%, 77.8%].
*Underlying pathophysiology*			
Chi et al. [86]	Genetic ancestry	LR; RF	HLA-DRB1*15:01 and HLA-DRB1*03:01 alleles.
Forbes et al. [87]	Gut microbiota	RF;	Accuracy in [82.0%, 84.0%];
			AUC in [91.0%, 94.0%].
*Improve measurement tools*			
Sébastien et al. [88]	Gait analysis	ET;	Accuracy in [70.9%, 91.7%].
Michel et al. [89]	Quality of life	DT; IRT;	Accuracy in [96.0%, 98.0%].
*Improve support groups*			
Rezaallah et al. [90]	Social media text	NLP; NB;	6 topics related to MS medication.
Deetjen et al. [91]	Text data	LR; NB;	Accuracy in [91.6%, 96.0%];
			56% informational and 44% emotional for MS.

Studies on diagnostic applications of ML to distinguish MS from other neurological diseases were less common, but they supplemented our list of promising diagnostic biomarkers of MS in the form of genomics and gut microbial data (Table 2 and Additional file 3). Four studies ($6.1\%$) worked at diagnostic applications of ML to distinguish MS from other neurological [47, 49] or medical diseases^3^ [46, 48]. These ML applications analyzed biological [46, 47, 49] or clinical data [48]. However, the ML accuracy of over 90.0% was reached only by analyzing gut microbial data through the LogitBoost classification algorithm [48].

Applications of ML to measuring MS status continued to encourage our search for disease biomarkers that can be measured more regularly and inexpensively than MRI (Table 2 and Additional file 3). ML was applied to measuring MS status through disability-scoring or severity level computing in eleven studies ($16.7\%$). Data analyzed by these applications were drawn from clinical [55, 58], physical [45, 50–52, 56, 59], physiological [53, 55, 57], and genetic [54] sources. However, the only applications to exceed the accuracy of 90.0% were those based on assessing body movements [53] or falls risk [52] using random forests and SVMs. In contrast, one included study concluded that falls risk should be incorporated into assessment of MS disease status [51].^4^ Interestingly, when considering longitudinal changes in progressive MS, the sensitivity^5^ of the *Combinatorial WeIght-adjuStEd * (CombiWISE) disability-scoring that integrates four clinical scales^6^ was consistently better than that of MRI [55].

ML applications to recognize MS sub-types or clinical-courses—such as RRMS, *Primary-Progressive MS * (PPMS), and SPMS, each of which might be mild, moderate, or severe—emphasized the role of medical records and omics data in the biomarker search (Table 2 and Additional file 3). MS sub-typing was addressed in seven studies (10.6%) by analyzing clinical [60–62] and biological [44, 63–65] data. However, the accuracy of over 90.0% was reported only when using data from medical records [62] or omics^7^ [64]. Again, decision trees and SVMs achieved the best ML performance.

In the same vein, ML applications were used to assess MS prognosis. SVMs to classify clinical data outperformed other algorithms and data sources with conclusions suggesting the incorporation of obesity and smoking history and status (Table 2 and Additional file 3). MS prognosis was studied in ten studies (15.2%) by analyzing clinical [66–71, 73, 74] and physiological [43, 66, 72] data. In this application category, only one study reported the 90.0% accuracy [74]: it used an SVM classifier on clinical data. Nevertheless, weaker evidence implicating obesity and smoking data as biomarkers of MS was provided in the context of applying the *Least Absolute Shrinkage and Selection Operator* (LASSO) algorithm to disability prediction [68].

Omics and physiological data, together with data from medical records, were promising when applying ML to the treatment of MS. Nine studies (13.6%) examined responses of PwMS to treatment (Table 3 and Additional file 3). These studies analyzed responses to drugs, including *interferon beta* (IFNb) [75, 78, 79, 81, 82], fingolimod [76, 80], natalizumab [77], and glatiramer acetate [83]. The *Area Under the receiver operating characteristic Curve* (AUC) reached over 90.0% only once [76]: this study classified *micro RiboNucleic Acid* (microRNA) data using random forests. Finally, after IFNb treatment, measuring heart rate^8^ [80] and triplet testing of *Caspase 2, Apoptosis-Related Cysteine Peptidase* (CASP2), *Interleukin 10* (IL10), and *Interleukin 12 Receptor Subunit Beta 1* (IL12Rb1) [75] were the strongest predictors for response to MS treatments.

The remaining studies contributed to our biomarker searching by looking at fatigue measurement and stressing the strengths of omics and gut microbiome data (Table 3 and Additional file 3). Four included studies (6.1%) targeted exacerbation of symptoms [84, 85] or underlying pathophysiology [86, 87]. Fatigue was a main source of impaired quality of life [84, 85], and certain genetic patterns^9^ were highly associated with PwMS [86]. In addition, particular patterns of gut microbial pathogens^10^ were found in MS [87]. Another four studies (6.1%) aimed to improve support groups for PwMS by using *natural language processing* (NLP) to explore online forum posts^11^ or patients’ experiences with MS medication [90, 91] or, alternatively, using decision-tree and extra-tree algorithms, to enhance measurement tools looking at walking patterns or quality-of-life assessments [88, 89].

### ML methods and ML datasets

To analyze the percentage of articles according to ML methods studies (details in Tables 2 and 3; and Additional file 3), an overview is presented in Fig. 3. Most included studies employed supervised ML algorithms (91.5%) and only a few proposed unsupervised solutions (4.6%). In the case of supervised ML, both classification algorithms [49.2%; incl., but not limited to, random forests and other decision trees (30.8%) as well as *K nearest neighbor* (KNN) and other KNN-type algorithms based on measuring the distance of, e.g., nearest neighbors (8.5%)] and regression algorithms [42.3%; incl., but not limited to, SVMs (15.4%) and logistic regression (10.8%)] were considered. Applications of later advancements in NNs (6.9%) were rare due to the limited amount of labelled paired input-output training data available for ML, the requirement to be able to justify its decision-making principles in healthcare, or slow adoption of these algorithms by researchers in medical informatics and decision-making. Our further breakdown (Fig. 4) implied that researchers considered decision trees, SVMs, regression models, NNs, and KNN-type ML algorithms for diagnosing PwMS. Usually, they used decision trees and SVMs for measuring disease status. Decision trees and regression algorithms were mostly considered for measuring responses to treatment and MS progression. Typically, all ML evaluation was conducted using hold-out methods in order to use all annotated data available for ML optimally.

As our quantitative analysis of ML algorithms, we reported the average AUC, accuracy, and F1 score from their performance evaluations with our findings shortlisting random forests and NNs among the best performing ML methods on the basis of their above 80% AUC.^12^ Most commonly, the included studies considered random forests with their average performance of the AUC of 89.9%, accuracy of 81.5%, and F1 score of 78.1%. In addition, NNs had the AUC of 81.3% and accuracy of 84.8%; SVMs had the accuracy of 79.7% and F1 score of 77.5%; and KNNs the accuracy of 76.8%; and decision trees the accuracy of 76.7%. Furthermore, 68% studies reported validation strategies including *k*-fold, leave-one-out, and nested cross-validation. Overall, most studies deployed supervised ML to predict future trends of MS, and ML models based on decision trees (i.e., random forests) performed the best and were most commonly used.

Clinical data were particularly useful sources for ML-based predictive models, but we identified room for exploring physiological and biological data as well for measuring MS prognosis and distinguishing between MS sub-types (Fig. 5). Clinical datasets — such as demographic data, *patient-reported outcomes* (PROs, i.e., direct responses from patients and controls), *clinician-assisted outcomes* (CAOs, i.e., responses provided via a clinician acting as intermediary), and *electronic medical records* (EMRs) — were used to separate PwMS from controls. PROs and CAOs could describe or reflect how a patient feels, functions, or survives while EMRs might be interrogated to extract demographic and clinical data including prescriptions, pathological diagnosis, medication usage, and so on. Researchers mostly used biological data to support MS diagnosis and to measure response to treatment (Fig. 6). Physiological (and physical) data were used in computer-assisted MS diagnosis and measurement of MS disease status. Predominantly clinical data were used for measuring MS prognosis, disease status, and distinguishing among MS sub-types.

Included studies considered both cross-sectional and time-series data from, for example, clinical, physiological, and biological sources, for purposes ranging from diagnosis and prognosis to measuring disease status and severity (Fig. 5). For the analyses, clinical data (34.5%) were most commonly used, followed by biological data (33.3%), and physical and physiological data (23.0%). These applications were typically siloed for each data type (e.g., natural language or biological signals), and multi-modal analyses had not been studied.

## Discussion

Overall, the included studies had many different purposes: most of them were developed to support the diagnosis of MS (30.3%; 20 out of 66), followed by measuring disease status (16.7%; 11/66), prognosis (15.2%; 10/66), response to treatments (13.6%; 9/66), and distinguishing MS sub-types (10.6%; 7/66), among others. Promising data sources in the search for MS biomarkers included medical records and other clinical data (e.g., medications, pathology, as well as clinical history and status); EEG, tremor, postural-sway, heart rate, and/or other physiological data; the EDSS, Scripps neurological rating scale, 25-foot walk, 9-hole peg test, and/or other disability-scoring data; genetics and/or other omics data; and gut microbiome and other biological data. The most promising biomarkers themselves consisted of measurements and evaluations of fatigue, stress, anxiety, depression, body movements, falls risk, inflammatory markers, disability, smoking variables, obesity, and/or inducing apoptosis.

However, most studies focused on one of these sources and biomarker types, and leads to potential drawbacks. For example, looking at studies investigating immunological markers [92–94], it is not surprising that mediators of inflammation such as cytokines [34] or genes associated with inflammation such as TNFSF10 [47] were predictive of MS versus non MS given the inflammatory nature of MS. The problem in general is to distinguish MS-related inflammation from other inflammatory aetiologies.

The majority of included studies focused on either diagnosis or prognosis without addressing treatment. These studies suggest that it might be possible to discover biomarkers for measuring MS status that are less invasive and expensive than MRI. However, bridging the gap between health science and data science calls for providing appropriate data resources and more holistic multimodal solutions to allow progress from classification to differentiate people living with and without MS, and/or measuring MS progression. That is, finding biomarkers to monitor treatment seems to be an understudied topic.

Our systematic review suggests that application of ML to the MS is yet to adopt the latest ML algorithms and to take full utility of these computational modelling methods which might support clinicians’ judgement and decision-making. Overall, we found that NNs, SVMs, and decision-tree based algorithms performed best at differentiating PwMS from controls and recognizing MS sub-types or clinical-courses. We believe this is explained by their tolerance for relatively small amounts of data to learn from and/or by ML researchers’ devotion to careful feature engineering [95, 96]. In general, applications of ML to MS are constrained by the limited amount of annotated data available and as a result, the latest advancements in deep NNs are yet to gain popularity. Another technical gap that we identified was the lack of time-series and longitudinal datasets to allow studying hidden Markov models, recurrent NNs, and other sequential ML methods.

One effective approach to facilitate progress should be to organize and facilitate the design, creation, release, and use of experimental protocols (e.g., guidelines for developing and reporting ML analyses in clinical research by [24] and [25]), shared datasets (e.g., MSBase [97] and MS Floodlight Open [98]), and other community resources (e.g., as part of shared tasks, computational challenges, evaluation campaigns, or hackathons such as the Intelligent Disease Progression Prediction at the 2022 Conference and Labs of the Evaluation Forum by Brainteaser [99] that targets amyotrophic lateral sclerosis and MS). Although the 66 included studies followed the cited guidelines carefully in their reporting, comparing their aims, outcomes, ML methods would benefit from shared experimental protocols, supported by more standardized evaluation. More widely in biomedical *natural language processing* (NLP), community initiatives of this kind with published problem specifications; training and test data; data processing, visualization, and evaluation code and software; and benchmark evaluations and lab overviews have been successful in establishing strong ecosystems across professions and disciplines to conceptualize clinically-meaningful problems and introduce ML methods that have become their new state-of-the-art solutions [100–104]. Their use has also enhanced replicability and reproducibility of biomedical research [105–108]. In addition, their use has facilitated transfer of technology to clinical practice [109] by viewing data as a holistic trustworthy source of information for clinical purpose [110].

We recognize two main limitations of this review. ML has been extensively applied to MRI, but this was deliberately excluded from the current study. In order to assess the possibility of finding an alternative to expensive, invasive, and time-consuming MRI. For recently-published reviews of ML application to MRI and its potential in clinical settings, see [18, 19]. Another limitation of the review was its exclusion of classical statistics algorithms. We refer the reader to the paper [111] for more information about the theoretical and experimental similarities and differences between these ML algorithms in the context of neuroscience.

Improving the capacity to differentiate RRMS from other subtypes of MS, and to rate disease severity and prognosis would significantly reduce the levels of uncertainty described by PwMS. This includes uncertainty related to future disease progression [13, 90, 91], whether to have children [92, 93], and fears of becoming a burden [94, 112]. However, alleviating uncertainty for some, might mean removing a source of hope that one’s condition might not be as severe as other people’s [95]. The capacity of ML to inform treatment decisions could therefore provide enormous benefit to PwMS whose current choices are often constitute a trade-off between potential side-effects and limited information about efficacy, making decisions difficult [96, 113].

The collection of adequate quantities of high-quality data requires engagement of PwMS, and a willingness on their behalf to participate, preferably over long periods of time to collect ongoing data. While the use of technology to monitor MS is becoming more common (e.g., smartwatch- and smart phone-based SmartMS Floodlight App [98]) [114], the use of these brings both benefits and costs to the wearer [15]. In particular, technology often requires frequent calibration [115–117], intrudes on daily activities [115, 116], and acts as a constant reminder of chronic health conditions [118]. While for scientists the benefits of having access to large quantities of data may be obvious, it is essential that we understand the implications for vulnerable users, such as PwMS [119, 120].

We believe ML has the potential to be very useful in the search for a non-MRI biomarker of MS if applied appropriately. To maximize the potential of ML in this way, we would suggest to expand the size of the data sets studied. For example, this can be facilitated by sharing of data between different centres and by soliciting direct involvement of PwMS through, e.g., open community resources and computational challenges. As part of them, extending the study of ML algorithms to the currently understudied deep learning and NNs in MS is advisable; out of the top-3 performing ML algorithms of NNs, decision trees, and SVMs (average accuracy of 84.8%, 81.5%, and 79.7%, respectively), NNs were deployed only in 6.9% of the 22 included studies while for the other two algorithms, this deployment rate was 30.8% and 15.4%, respectively.

## Conclusions

ML is applicable to determining how candidate biomarkers perform in the assessment of MS and its severity. For instance, the random forest algorithm is both a common and well-performing choice, whilst deep learning advances are yet to become prevalent. However, applying ML research to clinically meaningful problems, including developing decision-support tools to support clinicians to optimize diagnosis, treatment strategies, and analyze treatment responses in individual patients calls for creating appropriate data resources and shared experimental protocols. To illustrate, the progress of these health informatics applications seems to be hindered by insufficient quantity and quality of data. This calls for developing appropriate data resources to proceed from classification to clinically-meaningful differentiation of disease and enabling more holistic analyses across data modalities as opposed to segregated solutions for signal processing, natural language processing, and each other data type.

## Supplementary information

**Additional file 1**: Background on Machine Learning (PDF)

**Additional file 2**: Validity Evaluation Tables (Document)

**Additional file 3**: Detailed summary of the included papers (Excel)

**Additional file 4**: PRISMA 2020 Checklist (PDF)

**Additional file 5**: Search Results (Document)

**Additional file 6**:Generating Sunburst Plot - ML Applications

**Additional file 7**: Generating Sunburst Plot - ML Methods

## Data Availability

The data that support the findings of this study are all from the literature and can be found online. The specific articles are listed in Tables of the Results section above. Additionally, data generated from the analysis of the literature, as well as data and code to generate the Figures are available as an Excel spreadsheets, PDF, and Python scripts in the supplementary files and material.

## Abbreviations

AUC: Area under the receiver operating characteristic Curve
AI: artificial intelligence
CA: Candida Albicans
CASP2: Caspase 2, Apoptosis-related cysteine peptidase
CNS: Central nervous system
CAO: Cinician-assisted outcome
CombiWISE: Combinatorial weIght-adjuStEd
CRHR1: Corticotropin releasing hormone receptor 1
CXCR4: C-X-C motif chemokine receptor 4
EEG: Electroencephalogram
EMR: Electronic medical record
EC: Exclusion criterion
EDSS: Expanded disability status scale
GAN: Generative adversarial network
GM-CSF: Granulocyte-macrophage colony-stimulating factor
HLA-DRB1: Human leukocyte antigen haplotype, DR beta 1
IFNb: Interferon beta
IFN-$\gamma$: Interferon Gamma
IL2: Interleukin 2
IL10: Interleukin 10
IL12Rb1: Interleukin 12 Receptor Subunit Beta 1
KNN: K nearest neighbor
LASSO: Least absolute shrinkage and selection operator
ML: Machine learning
MRI: Magnetic resonance imaging
microRNA: Micro riboNucleic acid
MSFC: MS functional composite
MSSS: MS severity score
MS: Multiple sclerosis
NLP: Natural language processing
NN: Neural network
PRO: Patient-reported outcome
PwMS: People living with MS
PPMS: Primary progressive MS
PRISMA: Preferred reporting items for systematic reviews and meta-analyses
PROSPERO: Prospective register of systematic reviews
RRMS: Relapsing remitting MS
SPMS: Secondary progressive MS
SOM: Self-organizing maps
SVM: Support vector machine
TNF: Tumor necrosis factor
ligand: TNF
TNFSF10: SuperFamily, member 10

## Footnotes

1 Using an incognito window on Google Chrome to avoid personalized outcomes.

2 Namely, the *Tumor Necrosis Factor* (TNF), *Granulocyte-Macrophage Colony-Stimulating Factor* (GM-CSF), *Interferon Gamma* (IFN-$\gamma$), *Interleukin 2* (IL2), and/or *C-X-C Motif Chemokine Receptor 4* (CXCR4) [32]; *Corticotropin Releasing Hormone Receptor 1* (CRHR1) [33]; Ceramides [36]; *Candida Albicans* (CA) enzymes [44]; and/or velocity of index finger [37]

3 Namely, myalgic encephalomyelitis and chronic fatigue syndrome [46, 48] or juvenile idiopathic arthritis, stroke, colorectal cancer, and acquired immune deficiency syndrome [46]

4 Namely, fallers and near-fallers should be considered similarly in this measurement.

5 a.k.a. recall or true positive rate

6 Namely, the EDSS, Scripps neurological rating scale, 25-foot walk, and 9-hole peg test

7 Namely, transcriptomics or kynurenine pathway

8 Namely, baseline heart rates from fingolimod induced bradycardia

9 Namely, the *Human Leukocyte Antigen haplotype, DR beta 1* (HLA-DRB1) alleles HLA-DRB1*15:01 and HLA-DRB1*03:01

10 Such as Erysipelotrichaceae (higher) and Dialister (lower)

11 Namely, analyses of their emotional sentiments or informational contents

12 When a given ML method was considered in more than two included studies using the same performance evaluation measure(s), we averaged their respective measure values.

## References

[1] Reich DS, Lucchinetti CF, Calabresi PA (2018). Multiple sclerosis. New Engl J Med.

[2] Rotstein D, Montalban X (2019). Reaching an evidence-based prognosis for personalized treatment of multiple sclerosis. Nat Rev Neurol.

[3] Thompson AJ, Banwell BL, Barkhof F, Carroll WM, Coetzee T, Comi G, Correale J, Fazekas F, Filippi M, Freedman MS, Fujihara K, Galetta SL, Hartung HP, Kappos L, Lublin FD, Marrie RA, Miller AE, Miller DH, Montalban X, Mowry EM, Sorensen PS, Tintoré M, Traboulsee AL, Trojano M, Uitdehaag BMJ, Vukusic S, Waubant E, Weinshenker BG, Reingold SC, Cohen JA (2018). Diagnosis of multiple sclerosis: 2017 revisions of the McDonald criteria. Lancet Neurol.

[4] Karabudak R, Dahdaleh M, Aljumah M, Alroughani R, Alsharoqi IA, AlTahan AM, Bohlega SA, Daif A, Deleu D, Amous A, Inshasi JS, Rieckmann P, Sahraian MA, Yamout BI (2015). Functional clinical outcomes in multiple sclerosis: current status and future prospects. Multiple Sclerosis Related Dis.

[5] Gross RH, Sillau SH, Miller AE, Farrell C, Krieger SC (2019). The multiple sclerosis severity score: fluctuations and prognostic ability in a longitudinal cohort of patients with MS. Multiple Sclerosis J Exp Transl Clin.

[6] Meyer-Moock S, Feng Y-S, Maeurer M, Dippel F-W, Kohlmann T (2014). Systematic literature review and validity evaluation of the expanded disability status scale (EDSS) and the multiple sclerosis functional composite (MSFC) in patients with multiple sclerosis. BMC Neurol.

[7] Biomarkers Definitions Working Group (2001). Biomarkers and surrogate endpoints: preferred definitions and conceptual framework. Clin Pharmacol Ther.

[8] Ostmeyer J, Christley S, Rounds WH, Toby I, Greenberg BM, Monson NL, Cowell LG (2017). Statistical classifiers for diagnosing disease from immune repertoires: a case study using multiple sclerosis. BMC Bioinf.

[9] Brichetto G, Monti Bragadin M, Fiorini S, Battaglia MA, Konrad G, Ponzio M, Pedullá L, Verri A, Barla A, Tacchino A (2020). The hidden information in patient-reported outcomes and clinician-assessed outcomes: multiple sclerosis as a proof of concept of a machine learning approach. Neurol Sci.

[10] Jackson KC, Sun K, Barbour C, Hernandez D, Kosa P, Tanigawa M, Weideman AM, Bielekova B (2020). Genetic model of MS severity predicts future accumulation of disability. Ann Human Genet.

[11] Helland CB, Holmøy T, Gulbrandsen P (2015). Barriers and facilitators related to rehabilitation stays in multiple sclerosis: a qualitative study. Int J MS Care.

[12] Dennison L, McCloy Smith E, Bradbury K, Galea I (2016). How do people with multiple sclerosis experience prognostic uncertainty and prognosis communication?. Qual Study PLoS One.

[13] Dennison L, Yardley L, Devereux A, Moss-Morris R (2011). Experiences of adjusting to early stage multiple sclerosis. J Health Psychol.

[14] Desborough J, Brunoro C, Parkinson A, Chisholm K, Elisha M, Drew J, Fanning V, Lueck C, Bruestle A, Cook M, Suominen H, Tricoli A, Henschke A, Phillips C (2020). ‘It struck at the heart of who I thought I was’: a meta-synthesis of the qualitative literature examining the experiences of people with multiple sclerosis. Health Expect.

[15] Pétrin J, Donnelly C, McColl M-A, Finlayson M (2020). Is it worth it?: the experiences of persons with multiple sclerosis as they access health care to manage their condition. Health Expect.

[16] Samuel AL (1959). Some studies in machine learning using the game of checkers. IBM J Res Dev.

[17] Jordan MI, Mitchell TM (2015). Machine learning: trends, perspectives, and prospects. Science.

[18] Mateos-Pérez JM, Dadar M, Lacalle-Aurioles M, Iturria-Medina Y, Zeighami Y, Evans AC (2018). Structural neuroimaging as clinical predictor: a review of machine learning applications. NeuroImage Clin.

[19] Hemond CC, Bakshi R (2018). Magnetic resonance imaging in multiple sclerosis. Cold Spring Harbor Perspectives Med.

[20] Zhang Z, Sejdić E (2019). Radiological images and machine learning: trends, perspectives, and prospects. Comput Biol Med.

[21] Liberati A, Altman DG, Tetzlaff J, Mulrow C, Gøtzsche PC, Ioannidis JP, Clarke M, Devereaux PJ, Kleijnen J, Moher D (2009). The prisma statement for reporting systematic reviews and meta-analyses of studies that evaluate health care interventions: explanation and elaboration. J Clin Epidemiol.

[22] Angelini M, Ferro N, Larsen B, Müller H, Santucci G, Silvello G, Tsikrika T (2014). Measuring and analyzing the scholarly impact of experimental evaluation initiatives. Proc Comput Sci.

[23] Goodfellow IJ, Pouget-Abadie J, Mirza M, Xu B, Warde-Farley D, Ozair S, Courville A, Bengio Y. Generative adversarial networks. 2014.

[24] Luo W, Phung D, Tran T, Gupta S, Rana S, Karmakar C, Shilton A, Yearwood J, Dimitrova N, Ho TB (2016). Guidelines for developing and reporting machine learning predictive models in biomedical research: a multidisciplinary view. J Med Internet Res.

[25] Stevens LM, Mortazavi BJ, Deo RC, Curtis L, Kao DP (2020). Recommendations for reporting machine learning analyses in clinical research. Circul Cardiovasc Qual Outcomes.

[26] Ahmadi A, Davoudi S, Daliri MR (2019). Computer aided diagnosis system for multiple sclerosis disease based on phase to amplitude coupling in covert visual attention. Comput Methods Programs Biomed.

[27] Andersen S, Briggs F, Winnike J, Natanzon Y, Maichle S, Knagge K, Newby L, Gregory S (2019). Metabolome-based signature of disease pathology in ms. Multiple Sclerosis Related Dis.

[28] Bertolazzi P, Felici G, Festa P, Fiscon G, Weitschek E (2016). Integer programming models for feature selection: new extensions and a randomized solution algorithm. Eur J Oper Res.

[29] Broza YY, Har-Shai L, Jeries R, Cancilla JC, Glass-Marmor L, Lejbkowicz I, Torrecilla JS, Yao X, Feng X, Narita A (2017). Exhaled breath markers for nonimaging and noninvasive measures for detection of multiple sclerosis. ACS Chem Neurosci.

[30] Chase HS, Mitrani LR, Lu GG, Fulgieri DJ (2017). Early recognition of multiple sclerosis using natural language processing of the electronic health record. BMC Med Inf Decision Making.

[31] deAndrés-Galiana EJ, Bea G, Fernández-Martínez JL, Saligan LN (2019). Analysis of defective pathways and drug repositioning in multiple sclerosis via machine learning approaches. Comput Biol Med.

[32] Galli E, Hartmann FJ, Schreiner B, Ingelfinger F, Arvaniti E, Diebold M, Mrdjen D, van der Meer F, Krieg C, Al Nimer F (2019). Gm-csf and cxcr4 define a t helper cell signature in multiple sclerosis. Nat Med.

[33] Goldstein BA, Polley EC, Briggs FB, Van Der Laan MJ, Hubbard A (2016). Testing the relative performance of data adaptive prediction algorithms: a generalized test of conditional risk differences. Int J Biostat.

[34] Goyal M, Khanna D, Rana PS, Khaiboullina S, Rizvanov A, Baranwal M (2019). Computational intelligence technique for prediction of multiple sclerosis based on serum cytokines. Front Neurol.

[35] Lötsch J, Schiffmann S, Schmitz K, Brunkhorst R, Lerch F, Ferreiros N, Wicker S, Tegeder I, Geisslinger G, Ultsch A (2018). Machine-learning based lipid mediator serum concentration patterns allow identification of multiple sclerosis patients with high accuracy. Sci Rep.

[36] Loetsch J, Thrun M, Lerch F, Brunkhorst R, Schiffmann S, Thomas D, Tegder I, Geisslinger G, Ultsch A (2017). Machine-learned data structures of lipid marker serum concentrations in multiple sclerosis patients differ from those in healthy subjects. Int J Mol Sci.

[37] Perera T, Lee W-L, Yohanandan SA, Nguyen A-L, Cruse B, Boonstra FM, Noffs G, Vogel AP, Kolbe SC, Butzkueven H (2019). Validation of a precision tremor measurement system for multiple sclerosis. J Neurosci Methods.

[38] Prabahar A, Natarajan J (2017). Prediction of micrornas involved in immune system diseases through network based features. J Biomed Inf.

[39] Severini G, Straudi S, Pavarelli C, Da Roit M, Martinuzzi C, Pizzongolo LDM, Basaglia N (2017). Use of nintendo wii balance board for posturographic analysis of multiple sclerosis patients with minimal balance impairment. J Neuroeng Rehabilit.

[40] Telalovic JH, Music A (2020). Using data science for medical decision making case: role of gut microbiome in multiple sclerosis. BMC Med Inf Decision Making.

[41] Torabi A, Daliri MR, Sabzposhan SH (2017). Diagnosis of multiple sclerosis from eeg signals using nonlinear methods. Australasian Phys Eng Sci Med.

[42] Zhang L, Wang L, Tian P, Tian S (2016). Identification of genes discriminating multiple sclerosis patients from controls by adapting a pathway analysis method. PLoS One.

[43] Kiiski H, Jollans L, Donnchadha SÓ, Nolan H, Lonergan R, Kelly S, O’Brien MC, Kinsella K, Bramham J, Burke T (2018). Machine learning eeg to predict cognitive functioning and processing speed over a 2-year period in multiple sclerosis patients and controls. Brain Topogr.

[44] Saroukolaei SA, Ghabaee M, Shokri H, Badiei A, Ghourchian S (2016). The role of candida albicans in the severity of multiple sclerosis. Mycoses.

[45] Sun R, Hsieh KL, Sosnoff JJ (2019). Fall risk prediction in multiple sclerosis using postural sway measures: a machine learning approach. Sci Rep.

[46] Bang S, Yoo D, Kim S-J, Jhang S, Cho S, Kim H (2019). Establishment and evaluation of prediction model for multiple disease classification based on gut microbial data. Sci Rep.

[47] Guo P, Zhang Q, Zhu Z, Huang Z, Li K (2014). Mining gene expression data of multiple sclerosis. PloS one.

[48] Ohanian D, Brown A, Sunnquist M, Furst J, Nicholson L, Klebek L, Jason LA (2016). Identifying key symptoms differentiating myalgic encephalomyelitis and chronic fatigue syndrome from multiple sclerosis. Neurology (E-Cronicon).

[49] Ostmeyer J, Christley S, Rounds WH, Toby I, Greenberg BM, Monson NL, Cowell LG (2017). Statistical classifiers for diagnosing disease from immune repertoires: a case study using multiple sclerosis. BMC Bioinf.

[50] Azrour S, Piérard S, Geurts P, Van Droogenbroeck M. Data normalization and supervised learning to assess the condition of patients with multiple sclerosis based on gait analysis. In: European Symposium on artificial neural networks, computational intelligence and machine learning (ESANN), 2014;649–654.

[51] Fritz NE, Eloyan A, Baynes M, Newsome SD, Calabresi PA, Zackowski KM (2018). Distinguishing among multiple sclerosis fallers, near-fallers and non-fallers. Multiple Sclerosis Related Dis.

[52] Gudesblatt M, Srinivasan J, Golan D, Bumstead B, Zarif M, Buhse M, Blitz K, Fafard L, Kantor D, Fratto T, et al. Machine learning models using multi-dimensional digital data and pros predict driving difficulties and falls in people with ms. In: MULTIPLE SCLEROSIS JOURNAL, 2019;vol. 25, pp. 342–343. Sage publications LTD 1 OLIVERS YARD, 55 CITY ROAD, LONDON EC1Y 1SP, ENGLAND

[53] Haider D, Ren A, Fan D, Zhao N, Yang X, Tanoli SAK, Zhang Z, Hu F, Shah SA, Abbasi QH (2018). Utilizing a 5g spectrum for health care to detect the tremors and breathing activity for multiple sclerosis. Trans Emerg Telecommun Technol.

[54] Jackson KC, Sun K, Barbour C, Hernandez D, Kosa P, Tanigawa M, Weideman AM, Bielekova B (2020). Genetic model of ms severity predicts future accumulation of disability. Ann Human Genet.

[55] Kosa P, Ghazali D, Tanigawa M, Barbour C, Cortese I, Kelley W, Snyder B, Ohayon J, Fenton K, Lehky T (2016). Development of a sensitive outcome for economical drug screening for progressive multiple sclerosis treatment. Front Neurol.

[56] McGinnis RS, Mahadevan N, Moon Y, Seagers K, Sheth N, Wright JA, DiCristofaro S, Silva I, Jortberg E, Ceruolo M (2017). A machine learning approach for gait speed estimation using skin-mounted wearable sensors: from healthy controls to individuals with multiple sclerosis. PloS one.

[57] Morrison C, Huckvale K, Corish B, Banks R, Grayson M, Dorn J, Sellen A, Lindley S (2018). Visualizing ubiquitously sensed measures of motor ability in multiple sclerosis: reflections on communicating machine learning in practice. ACM Trans Interac Intell Syst (TiiS).

[58] Shahid AH, Singh M, Kumar G (2019). Severity classification of multiple sclerosis disease: a rough set-based method. Int J Innov Technol Explor Eng.

[59] Supratak A, Datta G, Gafson AR, Nicholas R, Guo Y, Matthews PM (2018). Remote monitoring in the home validates clinical gait measures for multiple sclerosis. Front Neurol.

[60] Acquarelli J, Bianchini M, Marchiori E, et al. Discovering potential clinical profiles of multiple sclerosis from clinical and pathological free text data with constrained non-negative matrix factorization. In: European conference on the applications of evolutionary computation, 2016;pp. 169–183. Springer

[61] Fiorini S, Verri A, Tacchino A, Ponzio M, Brichetto G, Barla A. A machine learning pipeline for multiple sclerosis course detection from clinical scales and patient reported outcomes. In: 2015 37th Annual International Conference of the IEEE engineering in medicine and biology society (EMBC), 2015;pp. 4443–4446. IEEE 10.1109/EMBC.2015.7319381 26737281

[62] Gronsbell JL, Cai T. Semi-supervised approaches to efficient evaluation of model prediction performance series b statistical methodology. 2018. 10.1111/rssb.12502 PMC9586151 36275859

[63] Gupta M, Martens K, Metz LM, de Koning AJ, Pfeffer G (2019). Long noncoding rnas associated with phenotypic severity in multiple sclerosis. Multiple Sclerosis Related Dis.

[64] Lim CK, Bilgin A, Lovejoy DB, Tan V, Bustamante S, Taylor BV, Bessede A, Brew BJ, Guillemin GJ (2017). Kynurenine pathway metabolomics predicts and provides mechanistic insight into multiple sclerosis progression. Sci Rep.

[65] Lopez C, Tucker S, Salameh T, Tucker C (2018). An unsupervised machine learning method for discovering patient clusters based on genetic signatures. J Biomed Inf.

[66] Bejarano B, Bianco M, Gonzalez-Moron D, Sepulcre J, Goñi J, Arcocha J, Soto O, Del Carro U, Comi G, Leocani L (2011). Computational classifiers for predicting the short-term course of multiple sclerosis. BMC Neurol.

[67] Brichetto G, Bragadin MM, Fiorini S, Battaglia MA, Konrad G, Ponzio M, Pedullà L, Verri A, Barla A, Tacchino A (2020). The hidden information in patient-reported outcomes and clinician-assessed outcomes: multiple sclerosis as a proof of concept of a machine learning approach. Neurol Sci.

[68] Briggs FB, Justin CY, Davis MF, Jiangyang J, Fu S, Parrotta E, Gunzler DD, Ontaneda D (2019). Multiple sclerosis risk factors contribute to onset heterogeneity. Multiple Slerosis Related Dis.

[69] Flauzino T, Pereira WLdCJ, Alfieri DF, Oliveira SR, Kallaur AP, Lozovoy MAB, Kaimen-Maciel DR, Maes M, Reiche EMV (2019). Disability in multiple sclerosis is associated with age and inflammatory, metabolic and oxidative/nitrosative stress biomarkers: results of multivariate and machine learning procedures. Metabolic Brain Dis.

[70] Pruenza C, Solano MT, Díaz J, Arroyo R, Izquierdo G (2019). Model for prediction of progression in multiple sclerosis. IJIMAI.

[71] Tacchella A, Romano S, Ferraldeschi M, Salvetti M, Zaccaria A, Crisanti A, Grassi, F. Collaboration between a human group and artificial intelligence can improve prediction of multiple sclerosis course: a proof-of-principle study. F1000Research, 2017;6. 10.12688/f1000research.13114.1 PMC5990125 29904574

[72] Yperman J, Becker T, Valkenborg D, Popescu V, Hellings N, Van Wijmeersch B, Peeters L. Machine learning analysis of motor evoked potential time series to predict disability progression in multiple sclerosis. BioRxiv, 772996. 2019. 10.1186/s12883-020-01672-w PMC7085864 32199461

[73] Zhao Y, Healy BC, Rotstein D, Guttmann CR, Bakshi R, Weiner HL, Brodley CE, Chitnis T (2017). Exploration of machine learning techniques in predicting multiple sclerosis disease course. PLoS One.

[74] Zhao Y, Brodley CE, Chitnis T, Healy BC. Addressing human subjectivity via transfer learning: An application to predicting disease outcome in multiple sclerosis patients. In: Proceedings of the 2014 SIAM International Conference on Data Mining, 2014;pp. 965–973. SIAM

[75] Baranzini SE, Madireddy LR, Cromer A, D’Antonio M, Lehr L, Beelke M, Farmer P, Battaglini M, Caillier SJ, Stromillo ML (2015). Prognostic biomarkers of ifnb therapy in multiple sclerosis patients. Multiple Sclerosis J.

[76] Ebrahimkhani S, Beadnall HN, Wang C, Suter CM, Barnett MH, Buckland ME, Vafaee F (2020). Serum exosome micrornas predict multiple sclerosis disease activity after fingolimod treatment. Mol Neurobiol.

[77] Fagone P, Mazzon E, Mammana S, Di Marco R, Spinasanta F, Basile MS, Petralia MC, Bramanti P, Nicoletti F, Mangano K (2019). Identification of cd4+ t cell biomarkers for predicting the response of patients with relapsing-remitting multiple sclerosis to natalizumab treatment. Mol Med Rep.

[78] Karim ME, Petkau J, Gustafson P, Tremlett H, Group TBS (2017). On the application of statistical learning approaches to construct inverse probability weights in marginal structural cox models: hedging against weight-model misspecification. Commun Stat Simul Comput.

[79] Kasatkin D, Bogomolov YV, Spirin N (2018). Steps to personalized therapy of multiple sclerosis: predicting safety of treatment using mathematical modeling. Zhurnal nevrologii i psikhiatrii imeni SS Korsakova.

[80] Li K, Konofalska U, Akgün K, Reimann M, Rüdiger H, Haase R, Ziemssen T (2017). Modulation of cardiac autonomic function by fingolimod initiation and predictors for fingolimod induced bradycardia in patients with multiple sclerosis. Front Neurosci.

[81] Üçer S, Kocak Y, Ozyer T, Alhajj R (2017). Social network analysis-based classifier (snac): a case study on time course gene expression data. Comput Methods Programs Biomed.

[82] Walter E, Deisenhammer F (2014). Socio-economic aspects of the testing for antibodies in ms-patients under interferon therapy in austria: a cost of illness study. Multiple Sclerosis Related Dis.

[83] Patrick MT, Raja K, Miller K, Sotzen J, Gudjonsson JE, Elder JT, Tsoi LC (2019). Drug repurposing prediction for immune-mediated cutaneous diseases using a word-embedding-based machine learning approach. J Invest Dermatol.

[84] Bhattacharya S, Ramos AGC, Kawsar F, Lane ND, Gionta LM, Manidis J, Silvesti G, Vegreville M. Monitoring daily activities of multiple sclerosis patients with connected health devices. In: Proceedings of the 2018 ACM International Joint Conference and 2018 international symposium on pervasive and ubiquitous computing and wearable computers, 2018;666–669.

[85] Papakostas M, Kanal V, Abujelala M, Tsiakas K, Makedon F. Physical fatigue detection through emg wearables and subjective user reports: a machine learning approach towards adaptive rehabilitation. In: Proceedings of the 12th ACM international conference on pervasive technologies related to assistive environments, 2019;475–481.

[86] Chi C, Shao X, Rhead B, Gonzales E, Smith JB, Xiang AH, Graves J, Waldman A, Lotze T, Schreiner T (2019). Admixture mapping reveals evidence of differential multiple sclerosis risk by genetic ancestry. PLoS Genet.

[87] Forbes JD, Chen C-Y, Knox NC, Marrie R-A, El-Gabalawy H, de Kievit T, Alfa M, Bernstein CN, Van Domselaar G (2018). A comparative study of the gut microbiota in immune-mediated inflammatory diseases-does a common dysbiosis exist?. Microbiome.

[88] Piérard S, Phan-Ba R, Van Droogenbroeck M. Machine learning techniques to assess the performance of a gait analysis system. In: European symposium on artificial neural networks, computational intelligence and machine learning (ESANN), 2014;419–424.

[89] Michel P, Baumstarck K, Loundou A, Ghattas B, Auquier P, Boyer L (2018). Computerized adaptive testing with decision regression trees: an alternative to item response theory for quality of life measurement in multiple sclerosis. Patient Pref Adherence.

[90] Rezaallah B, Lewis DJ, Pierce C, Zeilhofer H-F, Berg B-I (2019). Social media surveillance of multiple sclerosis medications used during pregnancy and breastfeeding: content analysis. J Med Internet Res.

[91] Deetjen U, Powell JA (2016). Informational and emotional elements in online support groups: a bayesian approach to large-scale content analysis. J Am Med Inf Assoc.

[92] Kehne JH (2007). The crf1 receptor, a novel target for the treatment of depression, anxiety, and stress-related disorders. CNS Neurol Dis Drug Targets.

[93] Arenas-Ramirez N, Woytschak J, Boyman O (2015). Interleukin-2: biology, design and application. Trends Immunol.

[94] Virdis A, Colucci R, Bernardini N, Blandizzi C, Taddei S, Masi S (2019). Microvascular endothelial dysfunction in human obesity: role of tnf-α. J Clin Endocrinol Metabol.

[95] Pestian J, Brew C, Matykiewicz P, Hovermale DJ, Johnson N, Cohen KB, Duch W. A shared task involving multi-label classification of clinical free text. In: biological, translational, and clinical language processing, 2007;97–104.

[96] Nagalla R, Pothuganti P, Pawar DS. Analyzing gap acceptance behavior at unsignalized intersections using support vector machines, decision tree and random forests. In: ANT/SEIT, 2017;pp. 474–481.

[97] Kalincik T, Butzkueven H (2019). The MSBase registry: informing clinical practice. Multiple Sclerosis.

[98] Midaglia L, Mulero P, Montalban X, Graves J, Hauser SL, Julian L, Baker M, Schadrack J, Gossens C, Scotland A, Lipsmeier F, van Beek J, Bernasconi C, Belachew S, Lindemann M (2019). Adherence and satisfaction of smartphone- and smartwatch-based remote active testing and passive monitoring in people with multiple sclerosis: Nonrandomized interventional feasibility study. J Med Internet Res.

[99] Brainteaser: Intelligent Disease Progression Prediction at the Conference and Labs of the Evaluation Forum (CLEF) — IDPP@CLEF 2022. https://brainteaser.health/open-evaluation-challenges/idpp-2022/, last Accessed on 1 March 2022. 2021.

[100] Demner-Fushman D, Elhadad N (2016). Aspiring to unintended consequences of natural language processing: a review of recent developments in clinical and consumer-generated text processing. Yearbook Med Inf.

[101] Huang C-C, Lu Z (2016). Community challenges in biomedical text mining over 10 years: Success, failure and the future. Brief Bioinf.

[102] Filannino M, Uzuner Ö (2018). Advancing the state of the art in clinical natural language processing through shared tasks. Yearbook Med Inf.

[103] Suominen H, Kelly L, Goeuriot L (2018). Scholarly influence of the conference and labs of the evaluation forum ehealth initiative: review and bibliometric study of the 2012 to 2017 outcomes. JMIR Res Protocols.

[104] Suominen H, Kelly L, Goeuriot L, Ferro N, Peters C (2019). The scholarly impact and strategic intent of CLEF ehealth labs from 2012 to 2017. Inf Retrieval Eval Changing World: Lessons Learnfrom 20 Years of CLEF.

[105] Névéol A, Cohen K, Grouin C, Robert A. Replicability of research in biomedical natural language processing: a pilot evaluation for a coding task. In: Proceedings of the Seventh International workshop on health text mining and information analysis, pp. 78–84. Association for computational linguistics, Austin, TX. 2016.

[106] Cohen KB, Xia J, Zweigenbaum P, Callahan T, Hargraves O, Goss F, Ide N, Névéol A, Grouin C, Hunter LE. Three dimensions of reproducibility in natural language processing. In: Proceedings of the Eleventh International conference on language resources and evaluation (LREC 2018). European language resources Association (ELRA), Miyazaki, Japan. 2018. PMC5998676 29911205

[107] Mieskes M, Fort K, Névéol A, Grouin C, Cohen K. Community perspective on replicability in natural language processing. In: Proceedings of the International Conference on Recent Advances in Natural Language Processing (RANLP 2019), pp. 768–775. INCOMA Ltd., Varna, Bulgaria. 2019.

[108] Digan W, Névéol A, Neuraz A, Wack M, Baudoin D, Burgun A, Rance B (2020). Can reproducibility be improved in clinical natural language processing? A study of 7 clinical NLP suites. J Am Med Inf Assoc.

[109] Velupillai S, Suominen H, Liakata M, Roberts A, Shah AD, Morley K, Osborn D, Hayes J, Stewart R, Downs J (2018). Using clinical natural language processing for health outcomes research: overview and actionable suggestions for future advances. J Biomed Inf.

[110] Williamson R. Process and purpose, not thing and technique: How to pose data science research challenges. Harvard data science review. 2020. https://hdsr.duqduq.org/pub/f2cllynw

[111] Ballard DH. Modular learning in neural networks. In: AAAI, 1987;279–284

[112] Ramamurthy V, Yamniuk AP, Lawrence EJ, Yong W, Schneeweis LA, Cheng L, Murdock M, Corbett MJ, Doyle ML, Sheriff S (2015). The structure of the death receptor 4-tnf-related apoptosis-inducing ligand (dr4-trail) complex. Acta Crystallographica Sect F: Struct Biol Commun.

[113] Razzouk R, Shute V (2012). What is design thinking and why is it important. Rev Educ Res.

[114] Friedman B, Kahn PH, Borning A, Huldtgren A. In: Doorn, N., Schuurbiers, D., van de Poel, I., Gorman, M.E. (eds.) Value sensitive design and information systems, pp. 55–95. Springer, Dordrecht, 2013.

[115] Rashotte J, Tousignant K, Richardson C, Fothergill-Bourbonnais F, Nakhla MM, Olivier P, Lawson ML (2014). Living with sensor-augmented pump therapy in type 1 diabetes: adolescents’ and parents’ search for harmony. Can J Diab.

[116] Pickup JC, Ford Holloway M, Samsi K (2015). Real-time continuous glucose monitoring in type 1 diabetes: a qualitative framework analysis of patient narratives. Diab Care.

[117] Iturralde E, Tanenbaum ML, Hanes SJ, Suttiratana SC, Ambrosino JM, Ly TT, Maahs DM, Naranjo D, Walders-Abramson N, Weinzimer SA, Buckingham BA, Hood KK (2017). Expectations and attitudes of individuals with type 1 diabetes after using a hybrid closed loop system. Diab Educ.

[118] Lawton J, Blackburn M, Allen J, Campbell F, Elleri D, Leelarathna L, Rankin D, Tauschmann M, Thabit H, Hovorka R (2018). Patients’ and caregivers’ experiences of using continuous glucose monitoring to support diabetes self-management: qualitative study. BMC End Dis.

[119] Ceuninck van Capelle Ad, Meide Hvd, Vosman FJH, Visser LH (2017). A qualitative study assessing patient perspectives in the process of decision-making on disease modifying therapies (dmt’s) in multiple sclerosis (ms). PLOS ONE.

[120] Henschke A, Desborough J, Parkinson A, Brunoro C, Fanning V, Lueck C, Brew-Sam N, Brüstle A, Drew J, Chisholm K (2021). Personalizing medicine and technologies to address the experiences and needs of people with multiple sclerosis. J Personal Med.

