# Supplementary material for: The role of machine learning in developing non-magnetic resonance imaging based biomarkers for multiple sclerosis: a systematic review
Source: BMC Med Inform Decis Mak. 2022 Sep 15;22:242. doi: 10.1186/s12911-022-01985-5 (PMC9476596; doi:10.1186/s12911-022-01985-5)
Supplement: Supplementary file 1 — Additional file 1: Background on Machine Learning (PDF) [file 12911_2022_1985_MOESM1_ESM.pdf]

Supplementary Material A: Background on Machine Learning

Referred to jointly as machine intelligence, *artificial intelligence* (AI) and ML were introduced in the 1950s. AI was introduced with the goal of allowing machines to form abstractions and concepts, perform *natural language processing* (NLP), and solve problems previously reserved for humans and, in so doing, to improve themselves in creativity, self-improvement, randomness, theory of computation, computers, and *neural networks* (NNs) [1], among others. In 2021, its scope was the theory and development of computer systems that perform mathematical computing, learning, reasoning, problem solving, decision making, visual perception, NLP (e.g., speech recognition and translation of language) and other tasks that normally require human intelligence [2]. In comparison, the definition of ML evolved from giving computers the ability to learn without being explicitly programmed [3] to a type of AI that enables them to independently initiate and execute learning when exposed to new data [2]. The field can be subdivided to *supervised ML* where computer algorithms make decisions based on a given set of labelled paired input-output training data and *unsupervised ML* where algorithms do not require labelled data to build models [4, 5, 6, 7, 8, 9].

The key problems addressed by supervised ML are classification, ranking, and regression. In *classification problems*, the aim is to predict which class (or classes) a given input belongs to; for example, differentiating people living with and without MS. In *ranking problems*, the aim is to generate a ranked order of inputs based on a given criterion such as rating individual participants according to MS-disease severity. In *regression problems*, the aim is to generate a value representing each input along a continuous variable with respect to a given criterion. The continuous variable could reflect, for example, an individual's response to a given treatment regimen using a scale from 0% to 100%. The most prominent supervised ML algorithms [8] introduced in the 1950s–1980s were perceptron, naïve Bayes, and nearest neighbour classifiers; these were used together with artificial NNs and genetic algorithms for classification, ranking, and regression. The 1990s saw the introduction of random (decision) forests, gradient boosting, and *support vector machines* (SVMs). Hidden Markov models and conditional random fields were simultaneously developed to classify sequential data in the 1960s–2000s. From 2010 onwards, the state-of-the-art supervised ML has been based on deep NNs. Deep NNs are successfully used in classification and regression tasks, including signal and NLP, among others, on both cross-sectional and time-series data. However, they rely on a very large number of labelled input-output pairs for their training and abilities to justify their decision-making principles are weaker when compared to the earlier algorithms.

The key problems addressed by unsupervised ML are clustering, input representation, dimensionality reduction, and latent variable modelling. In *clustering problems*, the aim is to organise input instances so that instances that are similar with respect to a given criterion group near each other but are separated from dissimilar instances. *Input representation problems* refer to the development of a model of the input data which can efficiently represent the dataset in a deterministic or probabilistic manner. *Dimensionality reduction problems* aim to generate an output dataset with a reduced number of dimensions compared to those in the input dataset without loss of important information. *Latent variable modelling problems* involve discovering hidden information related to observable variables in the data. Unsupervised learning problems can be tackled with both algorithms that are not based on NNs (e.g., *principal component analysis* (PCA), random forests, and k-means) and NN-based algorithms (e.g., autoencoders [10] and *self-organizing maps* (SOMs) introduced in the 1980s [10], deep belief nets from the 2000s [11], as well as *generative adversarial networks* (GANs) from 2014 [12]).

References

[1] McCarthy, J., Minsky, M.L., Rochester, N., Shannon, C.E.: A proposal for the Dartmouth summer research project on artificial intelligence, August 31, 1955. AI Magazine **27**(4), 12 (2006)

[2] NIH, U.S. National Library of Medicine: Medical Subject Headings 2021. https://meshb.nlm.nih.gov/, last accessed on 17 February 2021 (2021)

[3] Samuel, A.L.: Some studies in machine learning using the game of checkers. IBM Journal of Research and Development **3**(3), 210–229 (1959)

[4] Jordan, M.I., Mitchell, T.M.: Machine learning: Trends, perspectives, and prospects. Science **349**(6245), 255–260 (2015)

[5] Baştanlar, Y., Özuysal, M.: Introduction to machine learning. Methods in Molecular Biology **1107**, 105–128 (2014)

[6] Deo, R.C.: Machine learning in medicine. Circulation **132**(20), 1920–1930 (2015)

[7] Bzdok, D., Krzywinski, M., Altman, N.: Machine learning: a primer. Nature methods **14**, 1119–1120 (2017)

[8] Glaser, J.I., Benjamin, A.S., Farhoodi, R., Kording, K.P.: The roles of supervised machine learning in systems neuroscience. Progress in Neurobiology **175**, 126–137 (2019)

[9] Nichols, J.A., Chan, H.W.H., Baker, M.A.: Machine learning: applications of artificial intelligence to imaging and diagnosis. Biophysical reviews **11**(1), 111–118 (2019)

[10] Ballard, D.H.: Modular learning in neural networks. In: AAAI, pp. 279–284 (1987)

[11] Hinton, G.E., Osindero, S., Teh, Y.-W.: A fast learning algorithm for deep belief nets. Neural Computation **18**(7), 1527–1554 (2006)

[12] Goodfellow, I.J., Pouget-Abadie, J., Mirza, M., Xu, B., Warde-Farley, D., Ozair, S., Courville, A., Bengio, Y.: Generative adversarial networks. (2014)
