# Supplementary material for: The role of machine learning in developing non-magnetic resonance imaging based biomarkers for multiple sclerosis: a systematic review
Source: BMC Med Inform Decis Mak. 2022 Sep 15;22:242. doi: 10.1186/s12911-022-01985-5 (PMC9476596; doi:10.1186/s12911-022-01985-5)
Supplement: Supplementary file 2 — Additional file 2: Validity Evaluation Tables (Document) [file 12911_2022_1985_MOESM2_ESM.docx]

**Supplementary Material B: Validity Evaluation Tables**

**Validity Evaluation Table 1.** We evaluated each study with respect to the validity guidelines for developing and reporting machine learning predictive models in biomedical research (Lou et al., 2016, Tables 1-5 available at <https://www.jmir.org/2016/12/e323/>) that we synthesised as 11 criteria below. We did not consider the 12th criterion of “Unexpected results reported and discussed” because our preference was to avoid excluding studies based on this desirable but not mandatory guideline. Due to disciplinary differences in research reporting, the potential limitations of the model (i.e., the 11th criterion consisting of the assumed input and output data format; potential bias and generalisability of the data used in modelling; and optionally, potential pitfalls in interpreting the model) could be discussed as, for example, limitations of the study, future work, or study scope, we did not evaluate this criterion as strictly as the other 10 criteria but marked the evaluation relaxations by * (e.g., formatting assumptions reported implicitly or explicitly as part of the materials and their processing methods or discussion of limitations worded as future work). No study returned more than one No answer and the seven No answers in total were limited to the 10th (i.e., Clinical implications derived & reported) and 11th criterion (i.e., Limitations discussed). Consequently, we did not see them as basis for excluding studies from this systematic review of machine learning algorithms as a way to support the discovery of biomarkers that can be measured regularly and inexpensively using non-invasive and readily-accessible techniques. However, as an additional validity assessment activity, we evaluated each of the 7 studies with a No answer in accordance with the recommendations for reporting machine learning analyses in clinical research (Stephens et al., 2020, Fig. 2-5, available at <https://www.ahajournals.org/doi/10.1161/CIRCOUTCOMES.120.006556>) – see Validity Evaluation Table 2.

| Criterion Study | Nature of study reported | Clinical goal identified | Current practice & prediction accuracy of existing models reviewed | Target of prediction identified and how this may benefit the clinical goal | Clinical setting identified (modelling context) | Prediction problem defined | Data sources identified, including inclusion& exclusion criteria | Predictive model built & tested/ validated | Final model & performance reported | Clinical implications derived & reported | Limitations discussed |
| --- | --- | --- | --- | --- | --- | --- | --- | --- | --- | --- | --- |
| Ahmadi et al. [26] | Original Research | Yes | Yes | Yes | Yes | Yes | Yes | Yes | Yes | Yes | Yes* |
| Andersen et al. [27] | Original Research | Yes | Yes | Yes | Yes | Yes | Yes | Yes | Yes | Yes | Yes |
| Bertolazzi et al. [28] | Original Research | Yes | Yes | Yes | Yes | Yes | Yes | Yes | Yes | Yes | Yes* |
| Broza et al. [29] | Original Research | Yes | Yes | Yes | Yes | Yes | Yes | Yes | Yes | Yes | Yes* |
| Chase et al. [30] | Original Research | Yes | Yes | Yes | Yes | Yes | Yes | Yes | Yes | Yes | Yes |
| deAndres-G. et al. [31] | Original Research | Yes | Yes | Yes | Yes | Yes | Yes | Yes | Yes | Yes | Yes* |
| Galli et al. [32] | Original Research | Yes | Yes | Yes | Yes | Yes | Yes | Yes | Yes | Yes | Yes* |
| Goldstein et al. [33] | Original Research | Yes | Yes | Yes | Yes | Yes | Yes | Yes | Yes | Yes | Yes |
| Goyal et al. [34] | Original Research | Yes | Yes | Yes | Yes | Yes | Yes | Yes | Yes | Yes | No |
| Lotsch et al. [35] | Original Research | Yes | Yes | Yes | Yes | Yes | Yes | Yes | Yes | Yes | Yes |
| Lotsch et al. [36] | Original Research | Yes | Yes | Yes | Yes | Yes | Yes | Yes | Yes | Yes | Yes |
| Perera et al. [37] | Original Research | Yes | Yes | Yes | Yes | Yes | Yes | Yes | Yes | Yes | Yes |
| Prabahar et al. [39] | Original Research | Yes | Yes | Yes | Yes | Yes | Yes | Yes | Yes | Yes | Yes* |
| Severini et al. [40] | Original Research | Yes | Yes | Yes | Yes | Yes | Yes | Yes | Yes | Yes | Yes |
| Telalovic et al. [42] | Original Research | Yes | Yes | Yes | Yes | Yes | Yes | Yes | Yes | Yes | Yes |
| Torabi et al. [43] | Original Research | Yes | Yes | Yes | Yes | Yes | Yes | Yes | Yes | Yes | Yes |
| Zhang et al. [44] | Original Research | Yes | Yes | Yes | Yes | Yes | Yes | Yes | Yes | Yes | Yes* |
| Kiiski et al. [45] | Original Research | Yes | Yes | Yes | Yes | Yes | Yes | Yes | Yes | Yes | Yes |
| Saroukolaei et al. [46] | Original Research | Yes | Yes | Yes | Yes | Yes | Yes | Yes | Yes | Yes | No |
| Sun et al. [47] | Original Research | Yes | Yes | Yes | Yes | Yes | Yes | Yes | Yes | Yes | Yes |
| Bang et al. [48] | Original Research | Yes | Yes | Yes | Yes | Yes | Yes | Yes | Yes | Yes | Yes |
| Guo et al. [49] | Original Research | Yes | Yes | Yes | Yes | Yes | Yes | Yes | Yes | Yes | Yes |
| Ohanian et al. [50] | Original Research | Yes | Yes | Yes | Yes | Yes | Yes | Yes | Yes | Yes | No |
| Ostmeyer et al. [51] | Original Research | Yes | Yes | Yes | Yes | Yes | Yes | Yes | Yes | Yes | Yes |
| Azrour et al [52] | Original Research | Yes | Yes | Yes | Yes | Yes | Yes | Yes | Yes | Yes | Yes |
| Fritz et al. [53] | Original Research | Yes | Yes | Yes | Yes | Yes | Yes | Yes | Yes | Yes | Yes |
| Gudesblatt et al. [54] | Original Research | Yes | Yes | Yes | Yes | Yes | Yes | Yes | Yes | Yes | Yes |
| Haider et al. [55] | Original Research | Yes | Yes | Yes | Yes | Yes | Yes | Yes | Yes | Yes | Yes* |
| Jackson et al. [56] | Original Research | Yes | Yes | Yes | Yes | Yes | Yes | Yes | Yes | Yes | Yes* |
| Kosa et al. [57] | Original Research | Yes | Yes | Yes | Yes | Yes | Yes | Yes | Yes | Yes | Yes |
| McGinnis et al. [58] | Original Research | Yes | Yes | Yes | Yes | Yes | Yes | Yes | Yes | Yes | Yes |
| Morrison et al. [59] | Original Research | Yes | Yes | Yes | Yes | Yes | Yes | Yes | Yes | Yes | Yes |
| Shahid et al. [60] | Original Research | Yes | Yes | Yes | Yes | Yes | Yes | Yes | Yes | Yes | Yes* |
| Supratak et al. [61] | Original Research | Yes | Yes | Yes | Yes | Yes | Yes | Yes | Yes | Yes | Yes |
| Acquarelli et al. [62] | Original Research | Yes | Yes | Yes | Yes | Yes | Yes | Yes | Yes | Yes | Yes* |
| Fiorini et al. [64] | Original Research | Yes | Yes | Yes | Yes | Yes | Yes | Yes | Yes | Yes | No |
| Gronsbell et al. [65] | Original Research | Yes | Yes | Yes | Yes | Yes | Yes | Yes | Yes | Yes | Yes |
| Gupta et al. [66] | Original Research | Yes | Yes | Yes | Yes | Yes | Yes | Yes | Yes | Yes | Yes |
| Lim et al. [67] | Original Research | Yes | Yes | Yes | Yes | Yes | Yes | Yes | Yes | Yes | Yes |
| Lopez et al. [68] | Original Research | Yes | Yes | Yes | Yes | Yes | Yes | Yes | Yes | Yes | Yes* |
| Bejarano et al. [70] | Original Research | Yes | Yes | Yes | Yes | Yes | Yes | Yes | Yes | Yes | Yes |
| Brichetto et al. [71] | Original Research | Yes | Yes | Yes | Yes | Yes | Yes | Yes | Yes | Yes | Yes |
| Briggs et al [72] | Original Research | Yes | Yes | Yes | Yes | Yes | Yes | Yes | Yes | Yes | Yes |
| Flauzino et al. [73] | Original Research | Yes | Yes | Yes | Yes | Yes | Yes | Yes | Yes | Yes | Yes* |
| Pruenza et al. [74] | Original Research | Yes | Yes | Yes | Yes | Yes | Yes | Yes | Yes | Yes | Yes* |
| Tacchella et al. [75] | Original Research | Yes | Yes | Yes | Yes | Yes | Yes | Yes | Yes | Yes | Yes |
| Yperman et al. [76] | Original Research | Yes | Yes | Yes | Yes | Yes | Yes | Yes | Yes | Yes | Yes |
| Zhao et al. [77] | Original Research | Yes | Yes | Yes | Yes | Yes | Yes | Yes | Yes | Yes | Yes |
| Zhao et al. [78] | Original Research | Yes | Yes | Yes | Yes | Yes | Yes | Yes | Yes | Yes | Yes |
| Baranzini et al. [79] | Original Research | Yes | Yes | Yes | Yes | Yes | Yes | Yes | Yes | Yes | Yes |
| Ebrahimkhani et al. [80] | Original Research | Yes | Yes | Yes | Yes | Yes | Yes | Yes | Yes | Yes | No |
| Fagone et al. [81] | Original Research | Yes | Yes | Yes | Yes | Yes | Yes | Yes | Yes | Yes | Yes |
| Karim et al. [82] | Original Research | Yes | Yes | Yes | Yes | Yes | Yes | Yes | Yes | Yes | Yes |
| Kasatkin et al. [83] | Original Research | Yes | Yes | Yes | Yes | Yes | Yes | Yes | Yes | Yes | No |
| Li et al. [84] | Original Research | Yes | Yes | Yes | Yes | Yes | Yes | Yes | Yes | Yes | Yes |
| Ucer et al. [85] | Original Research | Yes | Yes | Yes | Yes | Yes | Yes | Yes | Yes | No | Yes* |
| Walter et al. [86] | Original Research | Yes | Yes | Yes | Yes | Yes | Yes | Yes | Yes | Yes | Yes |
| Patrick et al. [87] | Original Research | Yes | Yes | Yes | Yes | Yes | Yes | Yes | Yes | Yes | Yes* |
| Bhattacharya et al. [88] | Original Research | Yes | Yes | Yes | Yes | Yes | Yes | Yes | Yes | Yes | Yes* |
| Papakostas et al. [89] | Original Research | Yes | Yes | Yes | Yes | Yes | Yes | Yes | Yes | Yes | Yes* |
| Chi et al. [90] | Original Research | Yes | Yes | Yes | Yes | Yes | Yes | Yes | Yes | Yes | Yes* |
| Forbes et al. [91] | Original Research | Yes | Yes | Yes | Yes | Yes | Yes | Yes | Yes | Yes | Yes |
| Sebastien et al. [92] | Original Research | Yes | Yes | Yes | Yes | Yes | Yes | Yes | Yes | Yes | Yes |
| Michel et al. [93] | Original Research | Yes | Yes | Yes | Yes | Yes | Yes | Yes | Yes | Yes | Yes |
| Rezaallah et al. [94] | Original Research | Yes | Yes | Yes | Yes | Yes | Yes | Yes | Yes | Yes | Yes |
| Deetjen et al. [95] | Original Research | Yes | Yes | Yes | Yes | Yes | Yes | Yes | Yes | Yes | Yes |

**Validity Evaluation Table 2.** Validity assessment of the 7 studies with a No answer in Validity Table 1 in accordance with the recommendations for reporting machine learning analyses in clinical research (Stephens et al., 2020, Fig. 2-5, available at <https://www.ahajournals.org/doi/10.1161/CIRCOUTCOMES.120.006556>).

| Recommendation Study | Nature of study reported | Guideline by Lou et al. (2016) with “No” | Year | Machine learning study design (see Fig. 2 for the 6 criteria) | Data sources and preprocessing (see Fig. 3 for the 7 criteria) | Disposition of subjects and features included in analysis (see Fig.4) | Model training and validation (see Fig. 5 for the 6 criteria) |
| --- | --- | --- | --- | --- | --- | --- | --- |
| Goyal et al. [34] | Original Research | Limitations discussed | 2019 | Yes | Yes | Yes | Yes |
| Saroukolaei et al. [46] | Original Research | Limitations discussed | 2016 | Yes | Yes | Yes | Yes |
| Ohanian et al. [50] | Original Research | Limitations discussed | 2016 | Yes | Yes | Yes | Yes |
| Fiorini et al. [64] | Original Research | Limitations discussed | 2015 | Yes | Yes | Yes | Yes |
| Ebrahimkhani et al. [80] | Original Research | Limitations discussed | 2020 | Yes | Yes | Yes | Yes |
| Kasatkin et al. [83] | Original Research | Limitations discussed | 2018 | Yes | Yes | Yes | Yes |
| Ucer et al. [85] | Original Research | Clinical implications  derived & reported | 2017 | Yes | Yes | Yes | Yes |
