# Supplementary material for: The role of machine learning in developing non-magnetic resonance imaging based biomarkers for multiple sclerosis: a systematic review
Source: BMC Med Inform Decis Mak. 2022 Sep 15;22:242. doi: 10.1186/s12911-022-01985-5 (PMC9476596; doi:10.1186/s12911-022-01985-5)
Supplement: Supplementary file 5 — Additional file 5: Search Results (Document) [file 12911_2022_1985_MOESM5_ESM.docx]

**Supplementary Material E: Search Results**

**Search Query 01**: “Multiple Sclerosis” AND (“Machine Learning” OR “Machine Intelligence” OR “Deep Learning” OR “Decision Tree*” OR “Random Forest*” OR “Pattern Recognition” OR “Genetic Algorithm*” OR “Supervised Algorithm*” OR “Decision Support System*” OR “Evolutionary Computation*” OR “Neural Network*” OR “Support Vector Machine*” OR “Autoencoder*” OR “Deep Belief Network*” OR “Adversarial Network*” OR “Self Organizing Map*” OR “Self Organising Map*”)

**Search Query 02**: “Multiple Sclerosis” AND (“Machine Learning” OR “Machine Intelligence”)

**Search Query 03**: "Multiple Sclerosis" AND "Machine Learning"

**Search Date**: 24 Dec. 19

**Database**: PubMed ([**https://www.ncbi.nlm.nih.gov/pubmed/**](https://www.ncbi.nlm.nih.gov/pubmed/))

**Date**: 01 Jan 2014 to 30 Sept. 2019 (only humans; **Search Query 01**)

**Items**: 75

**Database**: Cochrane ([**https://www-cochranelibrary-com.virtual.anu.edu.au/**](https://www-cochranelibrary-com.virtual.anu.edu.au/))

**Date**: Jan 2014 - Sept. 2019 (all text, only trails, no reviews, no protocols; **Search Query 01**)

**Items**: 25

**Database**: Google Scholar (Incognito window on Google Chrome)

**Date**: 2014 to 2019 (Sort by relevance; **Search Query 01**)

**Items**: Top 100

**Database**: ScienceDirect ([**https://www-sciencedirect-com.virtual.anu.edu.au/search/advanced**](https://www-sciencedirect-com.virtual.anu.edu.au/search/advanced))

**Date**: 2014 to 2019 (Sort by relevance; **Search Query 02**; Article type: Research articles (272) and Book chapters (68))

**Items**: 340

**Database**: Scopus ([**https://www-scopus-com.virtual.anu.edu.au/search/form.uri?display=basic**](https://www-scopus-com.virtual.anu.edu.au/search/form.uri?display=basic) )

**Date**: 2014 to 2019 (**Search Query 02**; Article type: Open Access (57) and other (112))

**Items**: 169

**Database**: Web of Science

(<http://apps.webofknowledge.com/summary.do?product=WOS&parentProduct=WOS&search_mode=GeneralSearch&qid=3&SID=C4VAHiOIPP4xjhzqjty&&page=1&action=changePageSize&pageSize=50>)

**Date**: 2014 to 2019 (Sort by relevance; **Search Query 02**; ALL FIELDS)

**Items**: 179

**Database**: Lens (<https://www.lens.org/>)

**Date**: 01 Jan 2014 to 30 Sept. 2019 (**Search Query 02**)

**Items**: 160

**Database**: dblp

(<https://dblp.dagstuhl.de/search/publ?q=%22Multiple%20Sclerosis%22%20AND%20%22Machine%20Learning%22>)

**Search Date**: 24 Dec. 19

**Date**: 2014 to 2019 (Sort by relevance; **Search Query 03**)

**Items**: 4

**Total**: 75+25+100+340+169+179+160+4 =1052

**Exclusion Criteria**:

1. Duplicates were removed.
2. Publications that are not original full peer-reviewed papers (including, but not limited to, reviews, book chapters, surveys, and abstracts) were removed.
3. Papers that are not about people living with multiple sclerosis were removed.
4. Papers that are not about machine learning were removed.
5. Papers working solely on data from magnetic resonance imaging (MRI), optical coherence tomography, visual fields tests, and/or lumbar puncture were removed, because these examinations that are not routinely conducted as standard clinical tests for multiple sclerosis or not in line with our research goal of developing minimally invasive biomarkers.

**Exclusion**: 213 (Criteria#1) + 157 (Criteria#2) + 335 (Criteria#3) + 20 (Criteria#4) + 261 (Criteria#5) = 986

**Included**: 1,052 - 986 = 66
